# “I feel like my job is to give patients hope” - perspectives of Community Health Workers and employers in Iowa: a mixed methods study

**DOI:** 10.1186/s12913-025-12536-9

**Published:** 2025-03-15

**Authors:** Amanda M. Sursely, Debra Kazmerzak, William Appelgate, Laurie M. Walkner, Samra Hiros, Roger Hileman, Heidi Haines, Rima A. Afifi

**Affiliations:** 1 Department of Epidemiology, College of Public Health, University of Iowa, 145 N. Riverside Drive, S427 CPHB, Iowa City, IA 52242 USA; 2 University of Iowa Prevention Research Center for Rural Health, Iowa City, IA USA; 3 HealthTeamWorks, Des Moines, IA USA; 4 University of Iowa College of Public Health, Institute of Public Health Practice, Research, and Policy, Midwestern Public Health Training Center, Iowa City, IA USA; 5 Iowa Department of Health and Human Services, Office of Rural Health, Des Moines, IA USA; 6 Department of Community and Behavioral Health, University of Iowa College of Public Health, Iowa City, IA USA

**Keywords:** Community health workers, Mixed methods, Iowa, Scope of practice, Healthcare team

## Abstract

**Introduction:**

Community Health Workers (CHWs) play a crucial role in extending health services, particularly for people who are medically underserved. Despite efforts to expand CHW programs nationally and in Iowa, challenges persist in defining their roles and responsibilities. Few studies have considered the perspectives of both CHWs and CHW employers simultaneously.

**Methods:**

We conducted an exploratory sequential mixed-methods study, first involving key informant interviews with CHWs and employers, which then informed the development of surveys distributed to both populations. We performed thematic analysis of qualitative data and calculated descriptive statistics of quantitative data.

**Results:**

Key informant interviews were conducted with five CHWs and five employers. An additional 123 CHWs and 81 employers responded to the survey. From the interviews, we report six themes, including roles and responsibilities, interaction with the broader health care team, and support needed. Survey respondents reported 69 unique job titles, a wide range of populations served, and diverse training needs. Despite 93.6% (*n* = 102) of CHWs receiving on-the-job training, 48% (*n* = 52) indicated they would still benefit from more training to be effective in their roles. 46% (*n* = 21) of employers reported unstable funding as a major barrier to program implementation.

**Discussion:**

CHWs in Iowa felt supported and valued as members of the care team, yet challenges to growing the CHW workforce remain. Our findings highlight the need for continued role definition of the CHW workforce, as well as the need to establish more sustainable sources of funding to ensure the continuity and expansion of this health equity-enhancing workforce.

**Supplementary Information:**

The online version contains supplementary material available at 10.1186/s12913-025-12536-9.

## Introduction

The American Public Health Association defines a community health worker [CHW] as “A frontline public health worker who is a trusted member of and/or has an unusually close understanding of the community served.” [1]. CHW is an umbrella title that incorporates a range of trained community health professionals whose well-established roles [2] are critical aspects of enhancing health equity [3–5]. Globally, CHWs have often played a critical role in extending health services for populations that are medically underserved [4, 6, 7], but the US has been slower to integrate CHWs into the traditional model of care [8–10]. The US healthcare system has struggled to meet the needs of medically underserved communities, exacerbating inequities in health outcomes [11, 12]. Systematic literature reviews have provided evidence that CHWs are effective in improving health outcomes for a variety of conditions, including maternal and child health, diabetes, and cardiovascular disease, as well as addressing the social determinants of health, particularly for populations impacted by health disparities [13–16]. Reflecting this potential, CHW programs have expanded across the US, not only in healthcare systems but also within public health agencies and community-based organizations [8, 10, 17, 18], with professional organizations emerging to sustain this growth [9, 19].

Just as the US has been slower than many other countries to incorporate CHWs into the community and clinical care team within the US, Iowa has been slower than other states to consider and adopt this workforce. In 2016, the Iowa Chronic Care Consortium [ICCC, now known as HealthTeamWorks] began the first exploration among health care and community-based providers, government agencies and academia to ascertain the level of understanding, enthusiasm for, and planned adoption of this workforce. Then and now, there is no state-level infrastructure in place - such as state-level certification, a CHW network or association, or mechanisms to bill for CHW services or sustain the workforce - to support, regulate or promote utilization of CHWs. However, over the last few years, the landscape has begun to change in Iowa. The state pursued and secured federal funding to dramatically improve accessibility to available training programs and is creating additional public health training for CHWs.

With the increasing interest in Iowa, and the growing momentum to expand the CHW workforce nationally and globally [20–22], defining the scope of practice in the state is more important than ever [23, 24]. Due to the grassroots development of many CHW programs, the titles, roles, responsibilities, and workforce profiles differ dramatically across the US and globally depending on context [3, 4, 25, 26]. A variety of factors influence the heterogeneity of CHW programs globally including disease-specific CHW roles, lack of funding for CHW programs, inadequate supervision and support, lack of understanding of the powerful contributions of CHWs, lack of functional systems to incorporate CHWs, and tenuous linkages and poor integration with the health system [23, 27, 28]. Similar factors have been noted in CHW programs across the US [18, 24, 27, 29], guiding the work in Iowa. The literature also notes that CHW programs must be fit to context, and therefore may continue to look differently in different communities; policies and procedures that drive effective programmatic strategies in one location may not reflect the situation or context in another.

Thus far, several US states have risen to this call to action and conducted workforce analyses of community health work in their state [29–32], but this has yet to be done in Iowa. Additionally, few analyses have simultaneously considered the viewpoints of both CHWs and the individuals who employ and supervise them. The aim of this exploratory research study was to assess the scope of practice for CHWs in Iowa, and to identify both CHW and CHW employer perspectives on the barriers and facilitators to the work, thereby providing information for program managers and policy makers in the state. Results of the study can also inform the broader literature by highlighting points of convergence and divergence of experiences and perspectives between CHW and CHW employers.

## Materials and methods

### Study design

To capture the experiences of both CHW employers and CHWs, we utilized an exploratory sequential mixed methods design across two phases [33]. In Phase I, we conducted an exploratory descriptive qualitative research study, with the aim of broadly understanding the lived experience of CHWs and CHW employers [34]. To this end, Phase I consisted of 10 key informant interviews – with five CHWs and five CHW employers–which we conducted in parallel and analyzed separately. We then used the qualitative findings in conjunction with a literature review to inform the development of a cross-sectional survey that was distributed to CHWs and CHW employers in Phase II. The methods and results have been reported as delineated in the Standards for Reporting Qualitative Research [SRQR] and the Consensus-Based Checklist for Reporting of Survey Studies [CROSS] [35, 36] (Appendix A). This study was reviewed by the University of Iowa Institutional Review Board and was determined to be not human subjects research as it informed the development of programmatic initiatives at the State level.

### Qualitative methods (Phase I)

#### Data collection tool

Based on a review of the literature [26, 37–41], the research team developed a semi-structured interview guide which was then reviewed and revised by members of the Iowa CHW Alliance (Appendix B). Questions focused on understanding the scope of work of CHWs, barriers to engaging in community health work, and perceptions of the workforce as they fit into the broader healthcare team. We identified and prioritized a set of core questions, with the remainder of questions asked as time permitted. Interview guides are included in supplemental Appendix C.

#### Recruitment and data collection

We selected interview participants from a list compiled by the Iowa Chronic Care Consortium [ICCC], an organization that has been working since 2016 to expand CHW programs in Iowa. Our selection of CHWs was intentional, to ensure diversity in terms of location of work [e.g.: hospital, public health department] and population of focus [e.g., refugee, Black/African American]. We invited a purposeful diverse [age, sex, organization, race/ethnicity] sample of six CHW employers, and six CHWs from 12 unique organizations to participate, with five of each group accepting. We aimed to amplify diverse perspectives on the role of CHWs in Iowa by including both CHWs and employers in our sample.

Interviewers were part of the research team and included authors on this paper [AS, DK, LW, RA, WA], all were familiar with the interview guide and had discussed apriori how to deliver the questions in a standard manner. We conducted the interviews over zoom from May-June 2021, at a time that was convenient for both the interviewer and the interviewee and lasted approximately 30–45 min. We offered all participants a $50 gift certificate as compensation for their time. With the participants’ consent, the interviews were recorded, de-identified, and sent to REV.com for verbatim transcription of dialogue. Only the primary research team had access to the raw data, and all subsequent quotations were presented with anonymous subject identifiers. Data analysis began after all interviews had been completed.

#### Data analysis and reporting

Three members of the research team [RA, AS, RH] independently reviewed a single randomly selected transcript and deductively [using interview question areas] and inductively [added areas that arose] developed relevant codes for thematic analysis. The team discussed any differences in selected codes, and a consensus was reached for a final set of codes. A single member of the research team [AS] then used this final codebook deductively for the remainder of the transcripts. As coding occurred, any additional codes that arose were coded, reviewed by the original three researchers, and then searched for deductively in the previous interviews by AS. The codes were summarized into emergent themes using thematic analysis [42]. NVivo12 was used to apply these codes and themes to sections of text and to extract coded passages for further synthesis into themes [43]. The themes highlighted in the main text were chosen based on their perceived relevance, and as a best reflection of the study goals. For example, one of the study goals was to understand the roles and responsibilities of CHWs in Iowa and thus this theme was highlighted. In comparison, the theme “location of work tasks” emerged in the interviews, but was not directly applicable to the study question and thus was selected for the supplement. The same themes are reported in aggregate for both CHW and CHW employers as there were no clear emergent differences.

### Quantitative methods (Phase II)

#### Data collection tool

Utilizing knowledge gained from the key-informant interviews, and a literature review of existing CHW workforce surveys [26, 29, 44–48] the research team developed two web-based cross-sectional surveys: one for CHWs and another for CHW employers. From the existing CHW workforce surveys, we selected questions to suit the needs of the current assessment, intentionally including questions on topics that emerged as important in the key-informant interviews. For example, in the interviews, CHWs emphasized gaps in training (Appendix E), and thus training questions were found and included as a question block in the survey. Although we did not undertake a quantitative assessment of survey validity prior to its use, the question bank was reviewed by members of the Iowa CHW Alliance for it’s clarity, order, and appropriateness, as a qualitative assessment of content validity. The final version was then revised based on their comments. The final survey tools each had 62 questions including socio-demographics; and encompassed five domains of interest: (1) Workforce Employment Characteristics, (2) Training and Hiring, (3) Scope of Work/Populations Served, (4) Barriers, and (5) Perceptions of the CHW role. Full survey tools contained both multiple choice and free-response questions, and can be found in Supplementary Appendix D. The eligible target populations for the two surveys included all individuals in Iowa who self-identified as an employer of one or more CHWs (Survey 1, Appendix D), or as a CHW themselves (Survey 2, Appendix D). As one goal of the project was to understand the breadth of the workforce under the CHW umbrella, we did not limit responses to any one job title, role, or organization type.

#### Recruitment and data collection

To aid in reaching this population, the ICCC developed an extensive contact list containing email addresses of CHWs, CHW employers, and the Iowa CHW Alliance members, based on the programmatic work they have been doing since 2016. Although this list likely includes many of the CHWs and CHW employers in Iowa, to our knowledge it is not a complete sampling frame. An invitation letter was sent to everyone on the contact list along with an informational video that provided guidance on how to access and respond to the survey via the online survey platform Qualtrics© [49]. Additionally, other partnering organizations such as the Iowa Cancer Consortium forwarded the invitation to their full mailing list. We sent out invitations to complete the survey every Monday between April 18 and the end of May 2022. To take advantage of snowball sampling and expand the contact list, recipients of the invitation were encouraged to share it with others they knew. The CHW and CHW Employer survey remained open until June 10th, 2022. However, responses to the employer survey were relatively low, so we refielded the employer survey only between May 8–18, 2023. All survey responses were anonymous, and no personally identifiable information was collected. Data were kept confidential on a password protected computer accessible only to the study team. This study’s statistical methodology was purely descriptive, and no statistical testing was performed. Thus, we did not calculate an apriori sample size. Instead, in the discussion we highlight the limitations of the studies generalizability.

#### Data analysis and reporting

We analyzed all survey data using SAS V9.4. Due to the use of snowball sampling, an overall non-response rate was not possible to calculate as there is no clear denominator, but item level non-response rates ranged from 0 to 15% for the CHW employer survey, and 0–33% for the CHW survey. We checked the response IP addresses both within and between surveys for duplicates, and no evidence of multiple participation was found. We removed all responses with < 30% completion from the analysis to remove participants who did not provide information beyond the initial demographic questions. We did not make any assumptions or imputations for missing data. We computed descriptive statistics [IE frequencies and counts] for all relevant variables.

## Results

### Qualitative results

We conducted key informant interviews with five CHWs and five CHW employers, representing 12 different organizations across the state. Demographic and employment characteristics of the interviewees are presented in Table 1.

**Table 1:** Demographic and employment characteristics of the CHWs and employers participating in key informant interviews

	CHWs	Employers
**Gender** ^**a**^		
Female	4	5
Male	1	0
Non-binary	0	0
**Race/Ethnicity** ^**a**^		
White	3	5
Black	1	0
Latinx	1	1
**Workplace**		
Non-Profit	1	3
Community Based Organization	0	2
Community Health Center	0	1
Hospital	1	1
Public Health Department	2	1
Physician’s office	2	0
Other	1	0
**Geographic Scope**		
Statewide	1	-
County-specific	4	-
Urbanicity		
Urban	2	-
Rural	1	-
Mixed	2	-
**Length of time employed as/employing CHWs**		
< 1 year	1	0
1–2 years	3	0
2–5 years	0	3
6–10 years	0	1
> 10 years	1	1

^a^Results are reported here as to reflect the wording of the survey question. It is acknowledged that gender and sex, as well as Hispanic/LatinX are separate constructs, and that the designations here may conflate the two

We abstracted a total of 20 themes from the interviews. In what follows, we describe selected themes and associated exemplary italicized quotes. Quotes are attributed to CHW employers [E-CHW] or CHW [CHW] below. Supplementary Appendix E includes all the themes, codes, and relevant quotes.

#### Theme: roles/responsibilities

When asked about the roles and responsibilities of CHWs, interviewees described specific activities they may carry out on a day-to-day basis such as patient education. Beyond specific responsibilities, responses highlighted the unique role of CHWs in addressing the social determinants of health. Participants stated that CHWs are many things to many people, for example: “*The role of community health workers is as varied as the places that have them*.” [E-CHW3].

The role of CHWs as patient advocates was also highlighted. They use insider or lived experience to help health care providers gain a more complete understanding of clients’ lives, and to bridge the gap in cultural knowledge. Interviewees also provided inspirational statements about the roles and responsibilities of the CHWs; e.g. “*a role we see is commonly looking at serving people literally outside of the traditional four walls of healthcare and it is looking at defining what it means to challenge ourselves to provide*,* not patient centered*,* but person centered*,* person respectful*,* kindness and humanity along with whatever the services are that we provide*” [E-CHW5].

Four of the CHW and five of the CHW employer interviewees noted challenges in carrying out the roles and responsibilities of CHWs. These included overloaded schedules, needing to create boundaries to ensure work-life balance, language barriers, translation of medical terms, and the broad scope of the job. Emphasized by the timing of the survey, several challenges around COVID mitigation and vaccines were discussed, resulting from community trust issues with the larger healthcare system. It was noted that progress in communication with communities would be difficult without the presence of the CHWs, who are trusted members of the community.

#### Theme: health care team

Interviewees described the interactions of CHWs with the rest of the health care team, discussing how the role is integrated in their organizations. Interviewees described the seamless connection and interaction between the CHWs and other members of the team, as well as with other organizations providing services in their community. Generally, both CHWs and CHW employers described the role as unique within the larger health care team and indicated that the role was clearly defined and understood by all team members, for example: *“I understand what my job is and what my responsibilities are and I think for the most part everybody else here does also.”* CHW4.

#### Theme: perceptions of the CHW role

All of the CHWs noted the satisfying nature of serving others. CHW1 noted: “*How just kindness and attention and being like you… just that little time that I’m with them*,* just seeing them change right in front of me*.” The value of CHWs was well emphasized by their employers, and it was noted that without them clients would likely experience a gap in care. For example, E-CHW2 noted: “*I think honestly in our current world*,* I feel like community healthcare workers have become even more integral*. *And I know that as we’re looking forward to the future*,* we’re seeing huge increases in the Burma population that has been willing to get vaccinated following education and regular conversation. We went from like 90% of the Burma community saying*,* “No*,* that’s scary*,* I don’t wanna do that*,*” in early March to seeing probably like 60% of them already vaccinated now”.*

Whilst satisfying, the job is also stressful, as mentioned by 3 of the CHWs and 2 of the CHW employers. For example, E-CHW2 said: “*So that ends up being that one caseworker [CHW] who’s supporting that one client is gonna be navigating between all of these different social service organizations. They have to explain in detail*,* really complicated*,* often medical and or legal jargon*,* concepts that are completely unfamiliar*.” Three of the CHWs also mentioned feeling a sense of helplessness when they are not able to meet all the clients’ needs.

#### Theme: communities served

Four CHWs and four CHW employers specified the communities they served. These included refugee populations [Congolese, Burmese, Marshallese, Pacific Islanders], immigrant populations, Hispanic/Latino populations [including people with undocumented status], people funded by Medicare/Medicaid, low-income communities, vulnerable population groups, specialized populations, lower literacy level communities, and anyone who comes to their clinic. As one CHW employer stated: “*Really across the spectrum*,* across payers*,* we found patients wanting to partner with us. That’s been cool to see too*.”[E-CHW4].

#### Theme: funding

CHW employers mentioned the importance of and challenge of funding CHW programs. Many described funding the program through a patchwork of options, including governmental [state and national and local] as well as national NGOs or Foundations, local organizations/businesses, or GoFundMe pages. This patchwork process was challenging as funding sometimes ended abruptly requiring programs to scramble to figure out options. As noted by CHW1: “*We could have had like a great plan six months ago and had a good resource and everything was working*,* but then*,* funding stopped*,* or something decided to not continue and now we’re back to square one*.”

#### Theme: support needed by CHWs

Four CHWs and one CHW employer noted the importance of support from others on the health care team: supervisors, physicians, and others in their organization. The desire for interprofessional connection and support was also emphasized, highlighting the need for CHWs across Iowa to be connected. Lastly, four CHWs discussed the need for both additional resources, as well as the critical importance of having a network of resources to refer to. A CHWemployer nicely summarized the need for general support for CHWs: “*CHWs need people who are*,* like what we all need*,* they need people who are coaches*,* mentors*,* patient*,* can teach*,* help people evolve and grow and then as they get to new levels of learning and understanding*,* help them get to the next opportunity*.” [E-CHW5].

### Survey results: CHWs

We received a total of 123 survey responses from CHWs, representing 74 different organizations. After removing ineligible responses [< 30% of questions answered], 109 remained eligible for analysis. The population sample was geographically diverse, representing 42 of Iowa’s 99 counties. Roughly equal representation of rural, urban, and micropolitan CHWs were reported, approximately 1/3 working in each setting.

#### Workforce characteristics

CHWs surveyed were predominantly white [70.6%] and female [86.2%]. This sample was highly educated, with 92% reporting some level of post-secondary education. Full demographic data of survey respondents is presented in Table 2.

**Table 2:** Summary of the socio-demographics of CHWs and employers who responded to the survey

	CHWs		Employers	
	Frequency	Percent	Frequency	Percent
**Gender** ^**a**^				
Female	94	86.2	34	75.6
Male	9	8.3	9	20
Non-Binary	1	0.9	0	0
Missing	5	4.6	2	4.4
**Race/Ethnicity** ^**a**^				
American Indian or Alaskan Native	1	0.9	1	2.2
Asian	1	0.9	0	0
Black or African American	15	13.8	4	8.9
Hispanic or Latino/a	7	6.4	0	0
Middle Eastern or North African	1	0.9	0	0
Multiracial	2	1.9	1	2.2
Native Hawaiian or Pacific Islander	1	0.9	0	0
White	77	70.6	37	82.2
Missing	4	3.7	2	4.4
**Highest Educational Attainment**				
High school diploma or GED	4	3.7	0	0
Trade/Technical school	5	4.6	2	4.4
Some college, but no degree	16	14.7	0	0
2-year degree	24	22.0	4	8.9
4-year degree	39	35.8	12	26.7
Graduate level degree	17	15.6	25	55.6
Missing	4	3.7	2	4.4

^a^Results are reported here as to reflect the wording of the survey question. It is acknowledged that gender and sex, as well as Hispanic/LatinX are separate constructs, and that the designations here may conflate the two

Highlighting the breadth of the workforce, CHWs are identified by a wide range of job titles [*n* = 69], including Family Wellbeing Specialist, Patient Navigator, and Maternal Health Specialist. CHWs also reported working for a variety of organizational types, most commonly non-profit organizations, physician offices, and community-based organizations. On average, the respondents had been working as a CHW in the US for 4.5 years, with a range of 1 month to 36 years. The majority of CHWs who responded are employed full-time [*n* = 77, 87.5%], with only a small number working on a part-time or volunteer basis. Of the 79 CHWs who reported their annual income, wages ranged from less than $20,000 a year to more than $45,000.

#### Training and hiring

When asked about the hiring requirements for their current role, 66% of CHWs reported that their organization required prior education, certification, or a specific desired skillset from their applicants; 58% also reported that their role required a specific amount of prior experience. After beginning their roles, 93.26% of CHWs received additional training. Notably, 48% of CHWs say they would benefit from additional training to carry out their job responsibilities. CHWs listed the additional training they need. CHWs most commonly requested training related to the management of specific health issues (*n* = 10), and up-to-date information regarding new and changing resources (*n* = 9). Responses received are reported in Table 3.

**Table 3:** Additional training that CHWs reported would be helpful for their role

Topic^a^	*N*
Interpersonal communication	2
Health issues/Specific Disease topics (e.g., COVID-19, prenatal care, substance use)	10
Leadership Training	1
Self-care coping skills and work-life balance	2
Grant Writing	1
Client advocacy, counseling, or mentoring techniques	4
Cultural competence, diversity training, or language instruction	3
Updated trainings to stay up to date with current best practices and to learn about new resources as they come out	9
Job shadowing and instruction on day-to-day workflow	4
Other	2

^a^This was an optional question, with written-in answers. Responses were summarized and grouped into the following categories

#### Populations and health conditions served

**Fig. 1:**
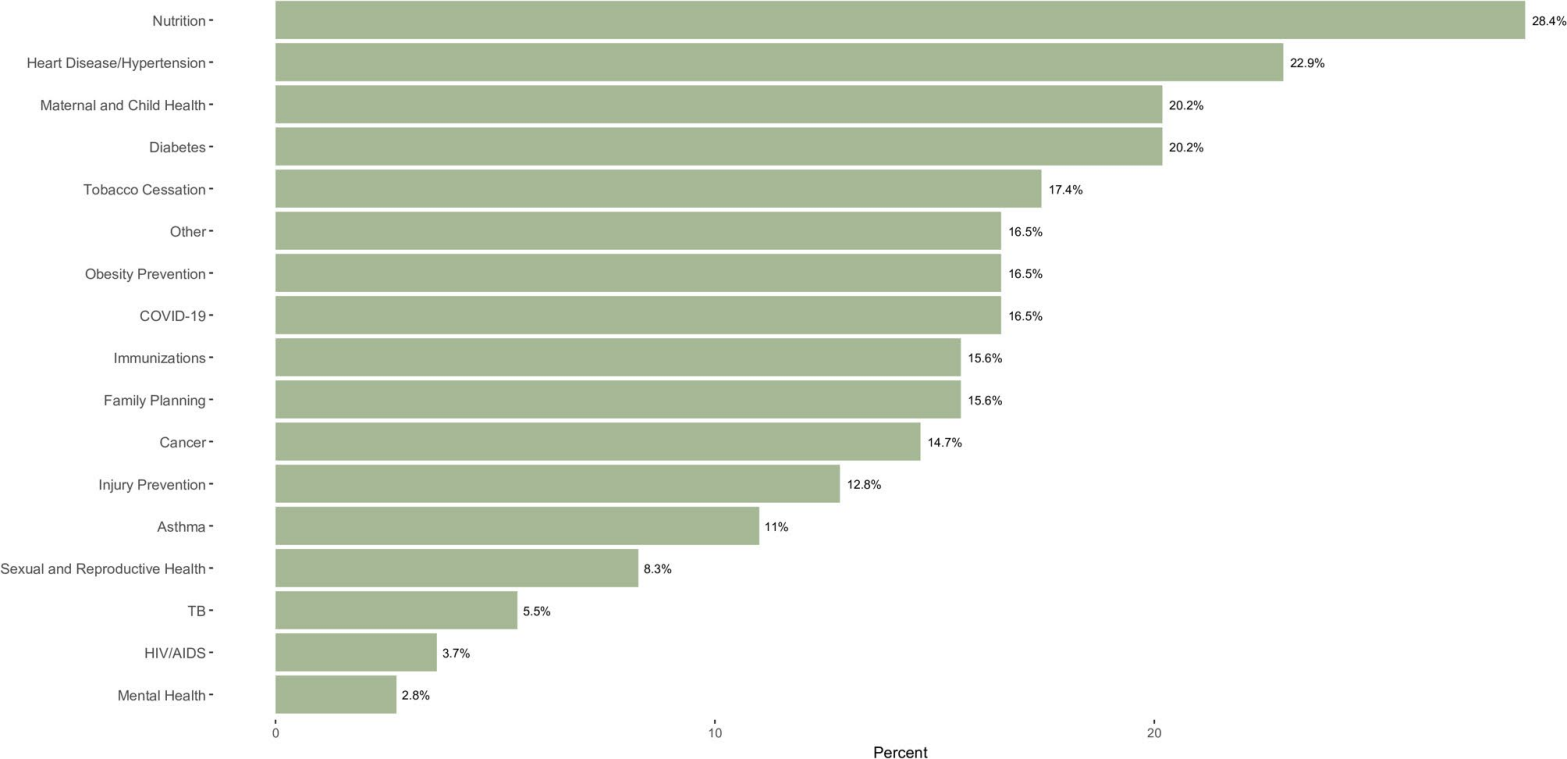
Health Conditions targeted by CHWs. Percentages amount to greater than 100% of respondents, as respondents could select all that applied

CHWs report working to provide services for a wide range of health conditions. Almost all respondents work with more than one health condition, and reported that no single condition is disproportionately targeted or neglected within the field (Fig. 1).

CHWs also provide services to individuals across the lifespan and serve a racially and ethnically diverse population, as shown in Table 4. About a third [28%] of CHWs indicated that they work with a specific immigrant or refugee population, and collectively reported that their clients speak 26 different languages, most commonly Spanish [*n* = 30, 27%], Swahili [*n* = 14, 12,8%], French [*n* = 10, 9.2%] and Karen [*n* = 6, 5.5%]. Despite the diversity of their clients’ primary languages, 80.46% of CHWs reported that they conduct their work entirely in English.

**Table 4:** Age group and race/ethnicity of the populations served by CHWs. Total N and % are greater than 121 and 100% respectively, as respondent could select all that applied

	Frequency	Percent
**Age Group of Individuals Served**		
< 18 years	10	6.8
18–24 years	15	10.3
25–39 years	22	15.1
40–59 years	28	19.2
60 + years	31	21.2
Families/all ages	40	27.4
**Race/Ethnicity of Individuals Served**		
American Indian or Alaskan Native	6	3
Asian	20	10
Black or African American	49	24.5
Hispanic or Latino/a	39	19.5
Middle Eastern or North African	9	4.5
Native Hawaiian or Pacific Islander	10	5
White	67	33.5

#### Perceptions of CHW work

Overwhelmingly, CHWs reported satisfaction in their roles, pride in their workforce, and the positive impact their work has on their communities. They feel supported by their supervisors and that they are a valued member of the care team within their organizations.

CHWs were asked about their biggest challenges in carrying out their work via a free-response question. The five most reported challenges were: (1) lack of funding and financial barriers, (2) availability of resources such as housing and transportation, (3) client follow-through and motivation for change, (4) language barriers, and (5) COVID-19.

### Results: CHW employers

We received a total of 81 responses from CHW employers. Of these, 45 were eligible for analysis and represented 41 organizations. Survey responses were deemed ineligible if < 30% of questions were answered.

#### Workforce characteristics

Of the CHW employers who responded, the majority were female [*n* = 34, 75%] and white [*n* = 37, 82.2%]. Over half of the CHW employers held a graduate level degree, and all had some sort of post-secondary education (Table 2).

Seventy percent of CHW employers indicated that their organizations began employing CHWs after the year 2020. These programs which are less than 3 years old make up approximately 40% of the total survey responses. All the programs have either maintained or increased the number of CHWs they employ, with plans of continued growth. The largest reported program currently employs 210 CHWs, and the smallest employs only one. Similarly, the number of clients served by these programs annually varies from < 100 to > 5,000.

The majority [*n* = 34, 75%] of programs employ at least some of their CHW’s full-time. Compensation is provided both via monthly salary [*n* = 31, 68.8%], and hourly wages [*n* = 11, 24.4%]. Ten CHW employers provided the pay-scale for their CHWs, which ranged from $27,000 a year to > 100,000.

#### Training and hiring

Hiring seems to be a challenge for CHW employers of CHWs- around half indicated that they have some trouble finding qualified applicants despite an increase in demand for the position. To gain insight into the type of CHW candidate’s that CHW employers are looking for, we asked them to identify three qualities or skills they look for when hiring CHWs. This was an open-ended question with no pre-defined categories. The top five qualities or skills CHW employers look for when hiring a CHW: (1) Excellent communication skills, including active listening, (2) Connection to the community they serve, and active member of that community, (3) Compassion, (4) Cultural Sensitivity, and (5) Knowledge and technical know-how.

Beyond the soft skills, CHW employers also reported whether they had other formal hiring requirements such as education, or licensure: 58% noted that CHW positions did not require any licensure or certification, and 73% stated that CHWs received additional training once hired.

#### Populations and health condition served

CHW employers report that their CHWs provide services for a wide range of health conditions. Almost all programs work with more than one health condition, and no single condition is disproportionately targeted or neglected within the field.

Fifty percent of CHW employers indicated that their CHWs work with specific immigrant or refugee populations, including Burmese, Congolese, Eritrean, Karen, Chinese, Mexican, Sudanese, Syrian, Afghan communities, and/or communities speaking Swahili, Arabic, French, Kirundi. Only 17.8% of CHW employers indicated that all their CHWs speak the same languages as the populations they serve.

#### Barriers to program implementation

When CHW employers were asked to identify the major barriers to implementing a CHW program, a lack of stable funding was the most frequently selected barrier, along with the inability to be reimbursed for the services they provide.

Following this, CHW employers were asked to elaborate on their funding sources and estimate what percentage of their funding came from various entities. These responses highlighted the patchwork funding that almost all organizations are working with. Across organizations, 39 separate funding sources were reported, with the average program relying on 2–3 sources. Forty-six percent of CHW employers indicated that their funding sources are unstable, and around 35% reported that they may not be able to fund CHWs in the future.

#### Convergent themes and divergent perceptions

The findings from our study highlighted both convergent themes and divergent perspectives between CHWs and CHW employers, as summarized in Table 5. Convergent findings included the recognition of the diverse scope of work for CHWs, the importance of skills like communication and cultural sensitivity in the hiring process, and the wide range of populations served by CHWs. There were no divergent themes or subcodes found in the qualitative results between CHWs and CHW employers. The only divergent perceptions were highlighted in the quantitative survey results which indicated that CHWs and CHW employers report their payment deliverance and pay scales differently shedding light on the complex dynamics of compensation and employment arrangements in the CHW workforce. They also differ on perceptions of training and populations served.

**Table 5:** Convergent themes/subcodes and divergent perceptions of CHWs and employers

**Convergent Findings (CHW and Employers agree)**			
Theme	Sub-code	Qualitative results	Quantitative results
Roles/Responsibilities	Diverse roles	“The role of community health workers is as varied as the places that have them.” [E-CHW3]	CHWs identified a wide variety of unique job titles (*n* = 69)
Training	Competencies	“…outside of her role here at (Organization name), she really is a female strong leader in that whole community.… And she just knows everybody, people trust her people believe in her.” [E-CHW1] “And another thing which is very important is to find a trusted person of the community. For example, the immigrants, wanting to do a team plan and make it successful. You need somebody who can really reach out to many people who is accepted among the people…” [CHW5]	When asked to list valued characteristics of a CHW, both CHWs and Employers emphasized (1) Communication and active listening (2) Client advocacy (3) Technical knowledge and (4) Cultural competency/Language
	Gaps	“I feel I do not get enough time to properly train our FSS before we throw a caseload on them to appease funders. I feel like training is one size fits all and that is not how we should approach it to ensure we do not have high turnover” [E-CHW3] “How to be a CHW, I understand how to interact with the people I am serving but there is not actual training to help us BE a CHW. Nothing telling us how our daily operation should flow or what resources are available and how to overcome barriers we encounter” [CHW, survey]	48% of CHWs say they would benefit from additional training in order to carry out their job responsibilities
Communities Served	Communities Served	“We work with multiple different problems, concerns, all sorts of different types of people, low-income, high-income, aged. We do actually 19 and up. We’ll take referrals for anybody in 19 and up. So it’s a diverse population.” [E-CHW3]	CHWs all selected that they work with more than one health condition, and no one condition was disproportionately targeted or neglected (Fig. 1). They also reported working with a diverse client population (Table 4).
	Client Barriers	“And then her (client), she is from Guatemala. So her first language is not Spanish. It’s her own native language from her tribe. So that even makes it harder for her.” [CHW2]	Only 17.8% of employers indicated that all of their CHWs speak the same languages as the populations they serve. Supporting this, only 18% of CHWs reported speaking a second language
Funding		“We could have had like a great plan six months ago and had a good resource and everything was working, but then, funding stopped, or something decided to not continue and now we’re back to square one.” [CHW1] “Without payment for services we can only provide limited services that come up with patients. We aren’t able to do much more than basic screenings which are done during a different paid encounter with the patient” [E-CHW4]	46% of employers disagreed that their funding sources are stable, and around 35% reported that they may not be able to fund CHWs in the future.
Perceptions of CHW Role	Satisfying	“The CHW is a valuable member of our team and is critical to our community outreach and work toward health equity and addressing SDoH” [E-CHW, survey] “This is a very rewarding and exciting position, one that did not exist a few years ago within the healthcare system. I look forward to continuing and growing in this position” [CHW, survey]	90% of CHWs and 97% of employers agree or strongly agree that CHWs are valued members of the care team.
	Clarity of role	“Better defining CHWs would be key. There are so many entities with staff doing similar roles it becomes confusing for the community and those being served” [E-CHW, survey] “Coworkers understanding my purpose and usefulness would help them to buy into making referrals and understanding the process” [CHW, survey]	
**Divergent Perceptions (CHWs and Employers Disagree)**			
Workforce Characteristics	Payment deliverance		70% of employers indicated that their employees are paid via a monthly salary, whereas only 19% of CHWs claimed the same
	Pay scale		CHWs reported pay scales ranging from $20,000 a year to more than $45,000, Employers reported $27,000 a year to > 100,000`
Training and Hiring	Hiring requirements		66% of CHWs reported that their organization had a requirement for prior education, certification, or a specific desired skillset from their applicants, where only 42% of employers noted the same
Populations Served	The diversity of populations being served		50% of employers indicated that their CHWs work with a specific immigrant or refugee population, only 28% of CHWs reported the same. 86% of employers reported that their CHWs served Hispanic clients, compared to only 20% of CHWs

## Discussion

Our study aimed to determine the scope of practice for CHWs in Iowa, and to explore the viewpoints of both community health workers [CHWs] and CHW employers regarding the facilitators and barriers to their work. Through a mixed-methods approach, we conducted key informant interviews and distributed surveys to capture a comprehensive understanding of the landscape. This study provides a novel comparison of CHW and CHW employer perspectives, capturing the workforce dynamic differently from studies considering these populations in isolation.

CHWs in our study had a variety of job titles and worked in a range of organizational types, similarly to CHWs in other studies [29, 30, 32, 50]. CHWs in our study felt that their role in the healthcare system was unique; which was echoed in a study that surveyed by CHWs across 869 zip codes in the US [50]. Similarly to CHWs in other States [30], they also noted that they were highly motivated by service to others. While the CHWs that responded to the survey were predominantly white and English-speaking, our survey sample was more diverse than the general Iowa population. Nevertheless, further efforts to recruit CHWs directly from the communities they serve could enhance linguistic and cultural connections, strengthening trust and effectiveness in community health work. The importance of this was emphasized by the CHWs in our interviews, who indicated that their lived experience and the trust they had with communities helped them do their work. The importance of cultural and community connection has been also echoed in other studies [29, 30, 50]. Challenges noted by the CHWs in our study was the dearth of resources to support their clients, a concern CHWs in other studies have also noted [29, 30, 32]. And similar to other studies [30], CHWs in our study were funded through multiple sources, making their jobs precarious. With respect to CHW employers in our study, all reported plans for continued growth of their CHW workforce, as compared to only 51% of employers in rural and urban settings of Nebraska [29]. Employers in our study and in Nebraska [29] noted a variety of different funding sources for CHWs.

The qualitative and quantitative results in our study, as well as the viewpoints of CHWs and CHW employers were often convergent, but as noted in Table 5, sometimes divergent. We found misalignment in feedback around employment arrangements and compensation between CHW employers and CHWs. Employers provided a much wider range of pay scale than the CHWs; for the survey, we included a broad definition of CHW. Though this approach captures the breadth and complexity of the workforce, it could have contributed to the wide range of responses, pay-scales, titles and responsibilities captured in this survey. Irrespective, the CHWs noted pay that ranged between $20,000-~$45,000. The US Bureau of Labor Statistics noted that the median salary for CHWs in 2023 was $48,200; suggesting that the pay scale in Iowa may be low [51]. This could impact job satisfaction and retention, and warrants further consideration of equitable compensation strategies [52–55].

As noted above, the findings related to payment and compensation call for a reevaluation of the financial support and incentives provided to CHWs. On January 1st 2024, a first step towards this went into effect with the introduction of the Centers for Medicare & Medicaid Services [CMS] Medicare Physician Fee Schedule Final Rule [PFS]. This policy will allow for Medicare reimbursement of monthly community health and navigation services, and twice a year social determinants of health assessments by certified CHWs [56]. With 35% of CHW employers in Iowa indicating that unstable funding may render them unable to employ CHWs in the future, finding sustainable financial models that support the vital work carried out by CHWs is critical to the future of the workforce [57, 58]. The creation of this new reimbursement structure is an exciting step forward in ensuring sustainability of the CHW workforce and will hopefully set the precedent for continued expansion moving forward.

CHW roles and competencies have long been recognized [2]. Similar to findings of CHW surveys in other States and across the US [29, 30, 32, 50], our findings highlight the heterogeneity in how these roles are implemented in Iowa, which may be critical as a starting point for planning and infrastructure development at the state level. The insights into the training and skill requirements highlighted in our study can inform the development of standardized training programs that align with the expectations of both CHW employers and CHWs in the state. Standardized training programs are being implemented in other States such as Massachusetts [59]. By addressing the need for additional training in areas such as cultural competence and disease-specific knowledge, we can enhance the preparedness of CHWs to serve their diverse communities effectively.

As noted in the introduction, this work was guided by, and results were shared back with the Iowa CHW Alliance, in which CHWs are one of the stakeholders represented. Further meetings of the CHW Alliance can explore these results further to gain greater understanding of the perspectives of CHW and CHW employers; and enhance communication between them. Areas of divergence can be explored and resolved; while areas of convergence can be expanded.

As a group, CHWs are not currently networked or connected in any formal way in Iowa. This data can be useful in laying the groundwork for the need of formal mechanisms to provide support, share information and offer guidance to this workforce. As a result, in-person CHW networking and learning workshops are set to begin in Fall 2024 and Spring 2025. Plans are also in place for the Iowa CHW Alliance to engage in strategic planning in Fall 2024, for which CHW and CHW employer input will be encouraged and solicited.

### Limitations of the study

While our study contributes valuable insights into the perspectives of both CHWs and CHW employers, there are certain limitations that should be considered. The study was conducted in a specific geographic region [Iowa] and may not be generalizable to the workforces in other states. Iowa is the 13th most rural state in the US, with 36% of the population living in rural areas according to the 2020 census. With approximately one-third of the CHW survey respondents working in rural communities, applying these results to more urban communities should be approached with caution. The composition of survey respondents, while intended to be representative of the state, might not encompass the full spectrum of CHWs and CHW employers in Iowa as there is no full sampling frame available. Due to the anonymity of the data, it is also not possible to know if the CHW employers and CHWs who responded are from the same organizations, limiting comparison between the surveys. Additionally, these surveys were conducted in the spring of 2022. Whilst they do reflect the scope of the workforce at that time, the presence of COVID-19 related work may be temporary and reflective of the time period. It is unclear how much of that work has and will continue as public health manages the after effects of the pandemic. Lastly, and specific to survey items, the survey conflated gender with sex; we have noted this in the footnotes of the Table 2.

### Implications for practice and policy

Our study illuminates the perspectives of both CHWs and CHW employers, revealing mostly convergent findings. The study emphasizes the importance of equitable compensation, sustainable funding mechanisms, and standardized training to support the diverse and vital roles of CHWs.

State level research such as this has been an instrumental part of support and expansion for CHWs in other states [60] which is reflected here, as this study laid the groundwork to continue efforts to secure state-level leadership and support infrastructure development for CHWs in Iowa. The state has led CHW training partnerships, explored and implemented ways to incorporate CHWs into existing programs (e.g. AmeriCorps Public Health program) and engaged in planning for development of specialized training for CHWs (e.g. youth mental health training for CHWs).

Given the findings on funding instability, further research is needed to understand the best mix of sustainable funding sources to support CHWs and the organizations that employ them. The University of Iowa is collaborating with the Iowa Department of Health and Human Services (Iowa HHS) to conduct a comprehensive analysis, engaging stakeholders through guided discussions to inform decision-making on the use of CHWs in Iowa by organizations. The goal of this work is to develop a multi-phase evaluation plan for CHW programs. Findings from this ongoing work will expand the understanding of the value CHWs bring to organizations and communities.

In essence, our study highlights the significant role CHWs play in improving health outcomes and health disparities and their experiences. Their deep understanding of community health needs and trusted relationships make them invaluable contributors to health equity efforts. Specific to Iowa, a mostly rural State, the critical role of CHWs in underserved rural settings has been noted [52]. In rural areas, CHWs face unique challenges, including limited healthcare infrastructure, geographic barriers, and socioeconomic disparities. This workforce is able to leverage their deep-rooted connections within the community to facilitate positive health outcomes and foster community resilience.

In conclusion, this study’s findings contribute to the ongoing dialogue surrounding the optimization of the CHW workforce, as efforts are made to integrate their valuable contributions into the broader healthcare landscape.

## Supplementary Information

Supplementary Material 1.

## Data Availability

The datasets presented in this article are not readily available because in small rural areas, data could be identifiable. Requests to access the datasets should be directed to Rima Afifi (rima-afifi@uiowa.edu) or Samra Hiros (samra-hiros@idph.iowa.gov).

## Abbreviations

CHW: Community Health Worker
ICCC: The Iowa Chronic Care Consortium
