# Supplementary material for: “I feel like my job is to give patients hope” - perspectives of Community Health Workers and employers in Iowa: a mixed methods study
Source: BMC Health Serv Res. 2025 Mar 15;25:386. doi: 10.1186/s12913-025-12536-9 (PMC11910000; doi:10.1186/s12913-025-12536-9)
Supplement: Supplementary file 1 — Supplementary Material 1. [file 12913_2025_12536_MOESM1_ESM.docx]

**Supplementary Appendix A**

Current CHW Alliance Members

Iowa Medicaid (Iowa HHS)

Iowa HHS (Co-Chair)

Wellpoint (formerly Amerigroup Iowa)

Area Substance Abuse Council

Broadlawns Medical Center

Central Iowa Healthworks

Connections Area Agency on Aging

Crescent Community Health Center

Dallas County Health Department

Delta Dental of Iowa Foundation

Des Moines University

Des Moines Area Community College

EMBARC

EveryStep

Grace Fitness

HealthTeamWorks, (formerly Iowa Chronic Care Consortium) Co-Chair

Iowa Pharmacy Association

Institute for Public Health Practice (UI College of Public Health)

Iowa Care Givers Association

Iowa Department on Aging

Bureau of Refugee Services (Iowa HHS)

Iowa Healthcare Collaborative

Iowa Primary Care Association

Iowa Total Care

Iowa Workforce Development

Linn County Public Health

Lutheran Services of Iowa

Mercy College of Health Sciences

MercyOne

Molina Healthcare of Iowa

One Iowa

Polk County Health Department

Prevent Child Abuse Iowa

Primary Health Care

Proteus

Siouxland Community Health Center

Telligen Community Initiative

United Way of Central Iowa

UnityPoint Health

University of Iowa Center on Disabilities and Development

CHP Community Hub

HRSA Region VII

**Supplementary Appendix B**

CHW and CHW employer interview guides

The following interview questions were used to guide the semi-structured, qualitative interviews conducted with CHWs and CHW employers. Highlighted were of particular importance, and prioritized when time was short. Interviewers were instructed to ask follow-up questions where needed and appropriate to garner a full and complete response.

Semi-Structured Interview Guide – CHW Employers

- Please tell me a little bit about yourself, your role in your organization and in what capacity you work with CHWs.
- What is the main reason that your organization decided to include CHWs as part of your services or care team?
- What are the primary roles and responsibilities of CHWs in your organization?
- How do they interact with others in your services or care team?
- What value do CHWs bring to your services or care team? Can you provide one example of that added value?
- Can you share a success story of a CHW in your organization?
- In what ways do CHWs help your organization accomplish its mission?
- How does your organization recruit individuals for CHWs positions?
- What qualities, and what skills do you look for in selecting a CHW?
- Thinking back to the individuals who have been most successful in their roles, what qualities, skills and characteristics have they possessed?
- Have you ever had a CHW who wasn’t a good fit for the role? What qualities contributed to the struggles they faced in the position?
- What communities/populations groups are most served by CHWs?
- Can you give an example of when your CHWs brought your attention to a priority issue in that community that you had not considered or known about?
- What types of challenges do CHWs encounter while working with clients/patients?
  - How are CHWs supported to address or overcome these challenges?
- Can you describe the supervision and support structure for CHWs?
- How does the CHW program fit into your organizational/corporate strategy for the future?
- If you had the opportunity to change or rework your program, what would you do differently, if anything?
- What are the barriers to expanding your CHW program?
- How can we better define the CHW role to prevent overlap among other healthcare providers?
- Is there anything else you think is important for us to know about the impact of CHWs on community health?

Semi-Structured Interview Guide – CHWs

- When did you begin work as a CHW? What motivated you to become a CHW?
- What would you consider to be your primary roles and responsibilities as a CHW?
- Can you walk us through a typical day in your work as a community health worker?
- What communities or community (or populations) do you serve as a CHW? Why is it important to you to serve this community/population?
  - What communities do you see as having the greatest unmet needs?
  - Where do you provide services to your community?
  - How does language impact your work?
- Can you describe how you first started working at your current organization?
- Are there barriers to entry for individuals in the community seeking to become a CHW?
- What training did you have prior to accepting your current position?
- Can you describe the training you got after you accepted the CHW job?
  - How adequately did this prepare you for your work as a CHW?
  - What areas do you feel you need more training in (skills and knowledge)?
- How different is your current day-to-day work from the job you were hired to do?
- How well defined is your scope of practice? How well is your work understood by your team?
- CHWs are part of a health services or care team. Who else is on the services or care team and how do you work together?
- What is the unique role of the CHW on the health care team? How is that role valued by others?
- Do you have the opportunity to network or connect with other CHWs in the area?
- What other organizations do you partner or connect with to get resources within the community?
- We know that CHWs can work with individuals/families in crisis, with overwhelming needs, or in very difficult situations. How does the supervision the CHW receives help guide them through these kinds of situations?
- What (other) supports do you need from your employer to help you be good/effective at your work? OR What resources do you wish you had available when you try to promote health in your community?
- Think about your experience working as a community health worker in your community. What gives you the most satisfaction or pride in your work?
- What non-monetary benefits have you gained from being a CHW?
- What are the primary challenges you face in your work as a CHW?
- What is the most unexpected experience you had in your work as a CHW?
- When you identify a need in the community/population you work with as a CHW, how are you able to advocate for them to the organization you work at?
- What would happen if you weren’t there to help your clients?
- What is your favorite thing about being a CHW?
- What is your least favorite thing about being a CHW?
- How can the work of CHWs be enhanced for you to be most effective?
- Is there anything else you think is important for us to know about the impact of CHWs on community health?

# Supplementary Appendix C:

# Employer and CHW survey tools

# Iowa Community Health Worker Employer Survey

We are inviting you to participate in a survey about Community Health Workers in Iowa. The Prevention Research Center (PRC) for Rural Health at the College of Public Health, University of Iowa and the Iowa Chronic Care Consortium (ICCC) are partnering to complete a workforce profile and analysis examining Community Health Workers (CHWs) in Iowa. This work is funded the Iowa Department of Public Health’s State Office of Rural Health (SORH).

Community Health Worker (CHW) is a broad term, which encompasses many different job titles and responsibilities. We are reaching out to you because you have been identified as someone who’s organization employs individuals who fit our case definition of a Community Health Worker, and we would love the opportunity to hear about your perspectives and experiences.

**Community Health Worker Definition**

“Community Health Workers (CHWs) are frontline health professionals essential to advancing public and population health. CHWs’ specific job titles, employers, roles, responsibilities and populations of focus vary widely. The central tenet -- CHWs are often trusted members of, or possess trusting relationships with, the communities they serve. This relationship with the community enables outreach to individuals and families through education, navigation, informal counseling, social support, care delivery and advocacy. CHWs effectively foster connectivity between health/social services, community services and individuals, especially for underserved and marginalized populations. CHWs often provide their services wherever the community member lives, works, plays or worships, in a culturally sensitive manner to inspire self-sufficiency and achieve better health”

This survey will take approximately 30 minutes. Your responses are anonymous, and you may skip any questions that you do not wish to answer. Thank you for your time and participation.

| Organization Name: |  | Job Title: |  |
| --- | --- | --- | --- |

| What is your gender? | | |  |  |
| --- | --- | --- | --- | --- |
| ☐ | Male | |  |  |
| ☐ | Female | |  |  |
| ☐ | Non-binary | |  |  |
| ☐ | Other (please specify): ________________ | |  |  |
|  |  | |  |  |
| What is your race or ethnicity? (Please select all that apply) | | |  |  |
|  | | |  |  |
| ☐ | | American Indian or Alaska Native | ☐ | Native Hawaiian or Pacific Islander |
| ☐ | | Asian | ☐ | Middle Eastern or North African |
| ☐ | | Black or African American | ☐ | White |
| ☐ | | Hispanic or Latino/a | ☐ | Other (please specify): ___________ |

| What is the highest level of education that you have completed? | | | |  |
| --- | --- | --- | --- | --- |
|  | | | |  |
| ☐ | Primary School | ☐ | Some college, but no degree | |
| ☐ | Some high school, but no diploma | ☐ | 2-year degree | |
| ☐ | High school diploma (or GED) | ☐ | 4-year degree | |
| ☐ | Trade/technical school | ☐ | Graduate level degree | |

| Are you a certified clinician in the US? | | If yes, list your title: | |  |
| --- | --- | --- | --- | --- |
| \| ☐ \| Yes \| ☐ \| Community Health Center \| \| --- \| --- \| --- \| --- \| | \| ☐ \| No \| ☐ \| Community Health Center \| \| --- \| --- \| --- \| --- \| | |  |  |

| What year did your organization start hiring CHWs? ___________________ | | | |
| --- | --- | --- | --- |
|  | | | |
| At that time, how many CHWs did you employ? __________________ | | | |
|  | | | |
| How many CHWs do you currently employ? _________________ | | | |
| What department do CHWs fall under within your organization? _________________ | | | |
|  | | | |
|  | | | |
| What is the current status of CHWs in your organization? | | | |
| ☐ | We currently employ CHWs |  |  |
| ☐ | We have employed CHWs in the past, but do not currently |  |  |
| ☐ | We may employ CHWs in the future, but do not currently |  |  |
| ☐ | We are not likely to employ CHWs in the future, and do not currently | |  |

| What are your plans regarding the engagement of CHWs? | | | |
| --- | --- | --- | --- |
| ☐ | Planning to increase the number of CHWs |  |  |
| ☐ | Neither planning to increase or decrease the number of CHWs |  |  |
| ☐ | Planning to decrease the number of CHWs |  |  |
| ☐ | Unsure | |  |

| Prior to hiring CHWs, were the job duties handled by another role? If yes, please indicate the position or positions that these responsibilities fell on. | | | |  |
| --- | --- | --- | --- | --- |
| ☐ | The work was not being done prior to hiring CHWs | ☐ | Social Workers | |
| ☐ | Nurses | ☐ |  | |
| ☐ | Physicians | ☐ |  | |

| Do you primarily hire CHWs internally or externally? | |
| --- | --- |
| ☐ | Internally |
| ☐ | Externally |
| ☐ | Both |

What job titles do you use to identify CHWs? Please list all.

|  |
| --- |
|  |

| What is the average number of years worked in your organization by CHWs? | | | |
| --- | --- | --- | --- |
| ☐ | < 1 year |  |  |
| ☐ | 1 – 2 years |  |  |
| ☐ | 3 – 4 years |  |  |
| ☐ | 5 years or more | |  |

| Please indicate the primary objective of the work CHWs do within your organization (select only one). | | | |  |
| --- | --- | --- | --- | --- |
|  | | | |  |
| ☐ | Access to care/services | ☐ | Primary Prevention | |
| ☐ | Community development | ☐ | Secondary Prevention | |
| ☐ | Direct care | ☐ | Tertiary Prevention | |
| ☐ | Intervention | ☐ | Other (please specify): ___________ | |

| *Which of the following best describes the status of the CHWS in your organization? | | |
| --- | --- | --- |
| ☐ | Paid, Full time |  |
| ☐ | Paid, Part time |  |
| ☐ | Unpaid / Volunteer |  |

| Do the services provided by the CHWs in your organization involve direct patient care? (ex/ screenings) | ☐ Yes | ☐ No |
| --- | --- | --- |

What health conditions are targeted or addressed by your program? (please mark all that apply)

| ☐ | Asthma | ☐ | Maternal and Child Health |
| --- | --- | --- | --- |
| ☐ | Cancer | ☐ | Nutrition |
| ☐ | COVID-19 | ☐ | Obesity prevention |
| ☐ | Diabetes | ☐ | Sexual and Reproductive Health |
| ☐ | Family planning | ☐ | Substance Use |
| ☐ | HIV/AIDs | ☐ | TB |
| ☐ | Heart Disease/Hypertension | ☐ | Tobacco Cessation |
| ☐ | Injury Prevention | ☐ | Other (please specify): _____________ |
| ☐ | Immunizations |  |  |

Approximately how many unique clients are served annually by the CHWs in your program?

| ☐ | 1 – 100 | ☐ | 751 – 1,000 |
| --- | --- | --- | --- |
| ☐ | 101 – 250 | ☐ | 1,001 – 2,500 |
| ☐ | 251 – 500 | ☐ | 2,501 – 5,000 |
| ☐ | 501 – 750 | ☐ | 5,001 + |

| Do your program’s CHWs speak the languages of those they serve? | ☐ Yes, all | ☐ Yes, some | ☐ No |
| --- | --- | --- | --- |

What are the primary languages spoken by the populations the CHWs serve?

|  |
| --- |

Which of the following racial/ethnic groups do the CHWs work with? (select all that apply)

| ☐ | American Indian or Alaska Native | ☐ | Native Hawaiian or Pacific Islander |
| --- | --- | --- | --- |
| ☐ | Asian | ☐ | Middle Eastern or North African |
| ☐ | Black or African American | ☐ | Other (please specify): _____________ |
| ☐ | Hispanic or Latino/a |  |  |

| Do they work with a specific immigrant or refugee population? (i.e. Sudanese, Congolese, Mexican, etc.) | ☐ No | ☐ Yes (please specify): _______________ |
| --- | --- | --- |

| Do the CHWs you hire live in / identify with the communities they serve? | ☐ Yes, all | ☐ Yes, some | ☐ No |
| --- | --- | --- | --- |

Do the CHWs in your organization work with any specific population groups? Please select all that apply.

| ☐ | Children / Adolescents | ☐ | Prisoners |
| --- | --- | --- | --- |
| ☐ | COVID Patients | ☐ | People living with HIV/AIDS |
| ☐ | Domestic violence victims | ☐ | Pregnant or postpartum women and infants |
| ☐ | Drug users | ☐ | Refugees |
| ☐ | Elderly populations / Seniors | ☐ | Sex workers |
| ☐ | Farm workers | ☐ | College Students |
| ☐ | Foreign nationals / immigrants | ☐ | Uninsured individuals |
| ☐ | Gender and sexual minorities | ☐ | N/A I work with all populations |
| ☐ | Homeless individuals | ☐ | Other (please specify): ___________ |
| ☐ | Individuals with disabilities | ☐ | Other (please specify): ___________ |
| ☐ | Parents/families |  |  |

**The following set of questions relate to the practices you use to hire and train your CHWs.**

| Are CHWs required to have any formal education prior to being hired or volunteering? | ☐ Yes | ☐ No | ☐ Unsure |
| --- | --- | --- | --- |
| Are any of the CHW positions required to be licensed/certified? (I.e. RN, MSW, etc.) | ☐ Yes | ☐ No | ☐ Unsure |

| If yes, please list what you require. | _______________ |
| --- | --- |

What are three qualities or skills you look for when hiring a person to do CHW work?

| 1. |
| --- |
| 2. |
| 3. |

| Once having been hired, does the new CHW receive additional training? | ☐ Yes | ☐ No |
| --- | --- | --- |

If yes, please select the topics they are trained on.

| ☐ | N/A I did not receive training | ☐ | Interpersonal Communication |
| --- | --- | --- | --- |
| ☐ | Client Advocacy | ☐ | Interview strategies |
| ☐ | Counseling / Mentoring Techniques | ☐ | Leadership Training |
| ☐ | Cultural Competency | ☐ | Management / Organizational Skills |
| ☐ | First Aid / CPR | ☐ | Making Referrals |
| ☐ | Fundraising / Grant Writing | ☐ | Public Health Issues / Specific Disease Topics |
| ☐ | Health Education Methods | ☐ | Record Keeping / Data Management |
| ☐ | Health Coaching | ☐ | Safety |
| ☐ | Home visits | ☐ | Other (please specify): _____________ |

Who conducts the trainings for your CHWs?

| ☐ | N/A , CHWs do not receive additional training | ☐ | Nutritionist |
| --- | --- | --- | --- |
| ☐ | CHW supervisor | ☐ | Other CHWs |
| ☐ | Doctor | ☐ | Outside Contractors |
| ☐ | Health Educator | ☐ | Psychologist |
| ☐ | Nurse | ☐ | Social Worker |
|  |  | ☐ | Other (please specify): _____________ |

Please specify the name and source of any specific curriculum or materials used for CHW training.

|  |
| --- |

**Funding**

What are the funding sources for your CHW program? Please provide your best estimate.

| **Percent of Total Funding** | **Agency Type** | **Agency** | | | |
| --- | --- | --- | --- | --- | --- |
| % | ☐ Federal Agency | ☐ CDC | ☐ HRSA | ☐ NIH | ☐ USDA |
| % | ☐ State Agency | ☐ Education | ☐ Human Services | ☐ State Health Department | ☐ Labor |
| % | ☐ Local agency/ government | Please specify: | | | |
| % | ☐ Private foundation | Please specify: | | | |
| % | ☐ Non-profit organization | Please specify: | | | |
| % | ☐ Other public funding | ☐ City | ☐ County | ☐ Multicounty | ☐ Regional group |
| % | ☐ Other sources | Please specify: | | | |
| **100%** | | | | | |

| Are the services provided by the CHWs eligible for reimbursement? | ☐ Yes | ☐ No |  |
| --- | --- | --- | --- |
| If yes, through which agencies? | ☐ Medicaid | ☐ Medicare | ☐ Private Health Insurance |
|  | ☐ ACO | ☐ MCO | ☐ Value- based contract |

**Compensation and Incentives**

Select the ways in which your organization pays for the services provided by the CHWs.

| ☐ | Hourly wage | ☐ | Hours of direct service |
| --- | --- | --- | --- |
| ☐ | Monthly salary | ☐ | Productivity bonus |
| ☐ | Reimbursement for mileage | ☐ | Number of clients seen |

Does the compensation differ by experience or training? If yes, can you share the pay range?

|  |
| --- |

Which of the following benefits do your CHWs (paid or unpaid) receive?

| ☐ | Child care | ☐ | Pension or retirement plan |
| --- | --- | --- | --- |
| ☐ | Educational leave | ☐ | Personal leave |
| ☐ | Health Insurance | ☐ | Sick leave |
| ☐ | Transportation/mileage reimbursement | ☐ | Tuition assistance |
| ☐ | Parking | ☐ | Vacation accrual |
|  |  | ☐ | Other (please specify): _____________ |

| If you have volunteer CHWs are their expenses reimbursed? | ☐ Yes | ☐ No |  |
| --- | --- | --- | --- |

**Effectiveness**

| Does your organization use CHWs to the extent that you think optimal? | ☐ Yes | ☐ No |
| --- | --- | --- |
| If no, please elaborate. |  | |

|  |
| --- |

| Does your organization conduct a formal evaluation to assess the success of your CHWs in meeting their primary objectives? | ☐ Yes | ☐ No | ☐Unsure |
| --- | --- | --- | --- |

If yes, who conducts this evaluation?

| ☐ | College or University personnel | ☐ | Private consultants |
| --- | --- | --- | --- |
| ☐ | Program Staff | ☐ | Other (please specify): _____________ |

Do you monitor or collect data on the following?

| ☐ | CHWs themselves | ☐ | Outcomes |
| --- | --- | --- | --- |
| ☐ | Clients / families served | ☐ | Policy |
| ☐ | Community | ☐ | Services |

What barriers / obstacles have you encountered in trying to implement a CHW program?

| ☐ | Lack of stable funding | ☐ | Lack of community support |
| --- | --- | --- | --- |
| ☐ | Lack of support from other health care workers | ☐ | The work is already being done in another role |
| ☐ | CHW services not reimbursable | ☐ | Shortage of qualified applicants |
| ☐ | Turnover | ☐ | Other (please specify): _____________ |

| **Statement** | Strongly Disagree | Disagree | Neutral | Agree | Strongly Agree |
| --- | --- | --- | --- | --- | --- |
|  | | | | | |
| CHWs are critical to achieving the mission of our organization | ☐ | ☐ | ☐ | ☐ | ☐ |
| The services provided by the CHWs meet the needs of clients | ☐ | ☐ | ☐ | ☐ | ☐ |
| CHWs provide valuable insights into the needs of the communities they serve | ☐ | ☐ | ☐ | ☐ | ☐ |
| It is important for CHWs to identify with or live in the communities they serve | ☐ | ☐ | ☐ | ☐ | ☐ |
| CHWs are valuable members of the care team | ☐ | ☐ | ☐ | ☐ | ☐ |
| The CHW role is clearly defined within our organization | ☐ | ☐ | ☐ | ☐ | ☐ |
| The CHW role is distinct from other organizational roles | ☐ | ☐ | ☐ | ☐ | ☐ |
| It is easy to find qualified applicants to fill the roles when we are hiring | ☐ | ☐ | ☐ | ☐ | ☐ |
| The demand for CHWs is increasing | ☐ | ☐ | ☐ | ☐ | ☐ |
| **Funding** |  |  |  |  |  |
| The funding sources for CHWs are stable | ☐ | ☐ | ☐ | ☐ | ☐ |
| We will be able to fund CHWs in the future | ☐ | ☐ | ☐ | ☐ | ☐ |
| Finding a different source of funding for CHWs is a priority | ☐ | ☐ | ☐ | ☐ | ☐ |

| Is there anything else you would like us to know about your experiences as a CHW that was not covered in this survey? |  |
| --- | --- |

|  |
| --- |
|  |
|  |

| Lastly, in an effort to aid us in reaching all individuals involved in CHW work, please list any organizations you think might benefit from engagement with this survey. |
| --- |

|  |
| --- |
|  |
|  |

# Iowa Community Health Workers Survey

We are inviting you to participate in a survey about Community Health Workers in Iowa. The Prevention Research Center (PRC) for Rural Health at the College of Public Health, University of Iowa and the Iowa Chronic Care Consortium (ICCC) are partnering to complete a workforce profile and analysis examining Community Health Workers (CHWs) in Iowa. This work is funded the Iowa Department of Public Health’s State Office of Rural Health (SORH).

Community Health Worker (CHW) is a broad term, which encompasses many different job titles and responsibilities. We are reaching out to you because you have been identified as someone who’s job duties fit our case definition of a Community Health Worker, and we would love the opportunity to hear about your perspectives and experiences.

**Community Health Worker Definition**

“Community Health Workers (CHWs) are frontline health professionals essential to advancing public and population health. CHWs’ specific job titles, employers, roles, responsibilities and populations of focus vary widely. The central tenet -- CHWs are often trusted members of, or possess trusting relationships with, the communities they serve. This relationship with the community enables outreach to individuals and families through education, navigation, informal counseling, social support, care delivery and advocacy. CHWs effectively foster connectivity between health/social services, community services and individuals, especially for underserved and marginalized populations. CHWs often provide their services wherever the community member lives, works, plays or worships, in a culturally sensitive manner to inspire self-sufficiency and achieve better health”

This survey will take approximately 30 minutes. Your responses are anonymous and you may skip any questions that you do not wish to answer. Thank you for your time and participation.

| Organization Name : |  | Job Title : |  |
| --- | --- | --- | --- |

| \| How would you best describe the area in which you complete most of your work? \| ☐ Urban \| ☐ Rural \| ☐ Mixed Urban/Rural \| \| --- \| --- \| --- \| --- \| | |
| --- | --- | --- | --- | --- | --- |
| In which county of Iowa do you complete most of your work as a CHW? If you work regionally or statewide, please indicate that instead. | |
| ☐ | County: _______________________________ |
| ☐ | Region: _______________________________ |
| ☐ | I work statewide |

| What is your age, in years? | ____________ |
| --- | --- |

| What is your gender? | | | | |  |
| --- | --- | --- | --- | --- | --- |
| ☐ | Male | |  |  |  |
| ☐ | Female | |  |  |  |
| ☐ | Non-binary | |  |  |  |
| ☐ | Other (please specify): ________________ | |  |  |  |
|  |  | |  |  |  |
| What is your race or ethnicity? (Please select all that apply) | | | | |  |
|  | | | | |  |
| ☐ | | American Indian or Alaska Native | ☐ | Native Hawaiian or Pacific Islander | |
| ☐ | | Asian | ☐ | Middle Eastern or North African | |
| ☐ | | Black or African American | ☐ | White | |
| ☐ | | Hispanic or Latino/a | ☐ | Other (please specify): ___________ | |

| What is the highest level of education that you have completed? | | | |  |
| --- | --- | --- | --- | --- |
|  | | | |  |
| ☐ | Primary School | ☐ | Some college, but no degree | |
| ☐ | Some high school, but no diploma | ☐ | 2-year degree | |
| ☐ | High school diploma (or GED) | ☐ | 4-year degree | |
| ☐ | Trade/technical school | ☐ | Graduate level degree | |

| Are you a certified clinician outside the US? | | If yes, list your title: | |  |
| --- | --- | --- | --- | --- |
| \| ☐ \| Yes \| ☐ \| Community Health Center \| \| --- \| --- \| --- \| --- \| | \| ☐ \| No \| ☐ \| Community Health Center \| \| --- \| --- \| --- \| --- \| | |  |  |

| If you are from outside the US, how long have you been living in the US? | ____________ |
| --- | --- |

Other than English do you speak any languages fluently? If yes, which languages?

|  |
| --- |

**The following questions are related to your current work as a CHW**

What type(s) of organization(s) do you work for as a CHW? Check all that apply.

| ☐ | Hospital | ☐ | Community Health Center |
| --- | --- | --- | --- |
| ☐ | Physician’s Office | ☐ | Department of Public Health |
| ☐ | Community based organization | ☐ | Religious organization |
| ☐ | Other (please specify): ________________ | ☐ | Non-profit organization |

| Which of the following best describes your employment status as a CHW? | ☐ Paid, Full-Time | ☐ Paid, Part-time | ☐ Unpaid / Volunteer |
| --- | --- | --- | --- |

| How many hours do you work as a CHW in a typical week, including both paid and unpaid work? Please provide your best estimate. | |  |
| --- | --- | --- |
| ☐ | 1 – 9 hours | |
| ☐ | 10 – 19 hours | |
| ☐ | 20 – 29 hours | |
| ☐ | 30 – 39 hours | |
| ☐ | 40 – 50 hours | |
| ☐ | 50 – 60 hours | |
| ☐ | 60 + hours | |

How are you compensated for your services as a CHW? Please select all that apply.

| ☐ | Hourly | ☐ | Per Member Per Month |
| --- | --- | --- | --- |
| ☐ | Monthly Salary | ☐ | Hours of Direct Service |
| ☐ | Reimbursement for Mileage | ☐ | N/A (Unpaid or Volunteer) |
| ☐ | Per Number of Patients Seen |  |  |

| How much are you paid for your work as a CHW in a typical year? Please provide your best estimate | |  |
| --- | --- | --- |
|  | |  |
| ☐ | Less than $20,000 | |
| ☐ | $20,001 to $25,000 | |
| ☐ | $25,001 to $30,000 | |
| ☐ | $30,001 to $35,000 | |
| ☐ | $35,001 to $40,000 | |
| ☐ | $40,001 to $45,000 | |
| ☐ | $45,001 or above | |

| For how many years have you been working as a CHW in the United States (including all full-time, part-time and volunteer experience)? | ____________ | |
| --- | --- | --- |
| On average, how many clients do you see in an average month? | ____________ | |
| On average, how many encounters with clients do you have in a given month? | ____________ | |
| On average, what is the timeframe that you will work with each individual client? | ____________ | |
| Do you live in the county where you do most of your CHW work? | ☐ Yes | ☐ No |

| What age group do you most often work with? (select all that apply) | | |
| --- | --- | --- |
|  | | |
| ☐ | Under 18 years |  |
| ☐ | 18-24 years |  |
| ☐ | 25-39 years |  |
| ☐ | 40- 59 years |  |
| ☐ | 60+ years of age |  |

Which of the following racial/ethnic groups do you primarily work with? Please select all that apply.

| ☐ | American Indian or Alaska Native | ☐ | Native Hawaiian or Pacific Islander |
| --- | --- | --- | --- |
| ☐ | Asian | ☐ | Middle Eastern or North African |
| ☐ | Black or African American | ☐ | Other (please specify): _____________ |
| ☐ | Hispanic or Latino/a |  |  |

| Do you work with a specific immigrant or refugee population? (i.e. Sudanese, Congolese, Mexican, etc.) | ☐ No | ☐ Yes (please specify): _______________ |
| --- | --- | --- |

What are the primary languages spoken by the population you serve?

|  |
| --- |
|  |

What is the primary language that you conduct your work in?

|  |
| --- |

Which of the following population groups do work with? Please select all that apply.

| ☐ | Children / Adolescents | ☐ | Prisoners |
| --- | --- | --- | --- |
| ☐ | COVID Patients | ☐ | People living with HIV/AIDS |
| ☐ | Domestic violence victims | ☐ | Pregnant or postpartum women and infants |
| ☐ | Drug users | ☐ | Refugees |
| ☐ | Elderly populations / Seniors | ☐ | Sex workers |
| ☐ | Farm workers | ☐ | College Students |
| ☐ | Foreign nationals / immigrants | ☐ | Uninsured individuals |
| ☐ | Gender and sexual minorities | ☐ | N/A I work with any population |
| ☐ | Homeless individuals | ☐ | Other (please specify): ___________ |
| ☐ | Individuals with disabilities | ☐ | Other (please specify): ___________ |
| ☐ | Parents/families |  |  |

Is your work as a CHW related to any of the following health issues? Please select all that apply.

| ☐ | Asthma | ☐ | Maternal and Child Health |
| --- | --- | --- | --- |
| ☐ | COVID-19 | ☐ | Nutrition |
| ☐ | Cancer | ☐ | Obesity prevention |
| ☐ | Diabetes | ☐ | Sexual and Reproductive Health |
| ☐ | Family planning | ☐ | Substance Use |
| ☐ | HIV/AIDs | ☐ | TB |
| ☐ | Heart Disease/Hypertension | ☐ | Tobacco Cessation |
| ☐ | Injury Prevention | ☐ | Other (please specify): _____________ |
| ☐ | Immunizations |  |  |

Where do you do most of your work as a CHW? Please select all that apply.

| ☐ | Clinic or Hospital | ☐ | Religious Center |
| --- | --- | --- | --- |
| ☐ | Community Center | ☐ | Schools |
| ☐ | Home visits | ☐ | Other (please specify): ___________ |
| ☐ | Organization’s Location |  |  |

How do you currently deliver services to your clients? Please select all that apply.

| ☐ | E-mail | ☐ | Telehealth (i.e. zoom or video chatting) |
| --- | --- | --- | --- |
| ☐ | Group sessions | ☐ | Texting |
| ☐ | In person visits | ☐ | Other (please specify): _______________ |
| ☐ | One-on-one |  |  |

How do you prefer to deliver services to your clients? Please select all that apply.

| ☐ | E-mail | ☐ | Telehealth (i.e. zoom or video chatting) |
| --- | --- | --- | --- |
| ☐ | Group sessions | ☐ | Texting |
| ☐ | In person visits | ☐ | Other (please specify): _______________ |
| ☐ | One-on-one |  |  |

What activities do you currently do as a CHW? Please select all that apply.

| ☐ | Case management | ☐ | Home visits |
| --- | --- | --- | --- |
| ☐ | Clinical Services | ☐ | Office work |
| ☐ | Community Organization | ☐ | Peer Education / Mentoring |
| ☐ | Counseling | ☐ | Presenting in schools / community centers etc. |
| ☐ | Fundraising or Grant Writing | ☐ | Support groups |
| ☐ | Health Education | ☐ | Translation/Interpreting |
| ☐ | Health screenings | ☐ | Other (please specify): _____________ |

What resources do you connect to the people you serve? Please select all that apply.

| ☐ | Child Care | ☐ | Income Assistance |
| --- | --- | --- | --- |
| ☐ | Education Assistance | ☐ | Legal Services |
| ☐ | Employment | ☐ | Translation/Interpretation |
| ☐ | Food Assistance | ☐ | Transportation |
| ☐ | Fuel Assistance | ☐ | Violence Prevention |
| ☐ | Health Care | ☐ | N/A I do not connect people to resources |
| ☐ | Health Insurance Enrollment | ☐ | Other (please specify): _____________ |
| ☐ | Housing |  |  |

What are some of the biggest barriers/challenges you face while doing your work?

|  |
| --- |
|  |

For the following set of statements, please indicate your level of agreement or disagreement with each by selecting the appropriate box.

| **Statement** | Strongly Disagree | Disagree | Neutral | Agree | Strongly Agree |
| --- | --- | --- | --- | --- | --- |
|  | | | | | |
| I am proud to be a CHW. | ☐ | ☐ | ☐ | ☐ | ☐ |
| I am confident in the quality of the services I provide. | ☐ | ☐ | ☐ | ☐ | ☐ |
| Being a CHW gives me a sense of purpose. | ☐ | ☐ | ☐ | ☐ | ☐ |
| I feel valued as a member of the care team | ☐ | ☐ | ☐ | ☐ | ☐ |
| I have a positive impact on the community I serve. | ☐ | ☐ | ☐ | ☐ | ☐ |
| I have opportunities to network with other CHWs | ☐ | ☐ | ☐ | ☐ | ☐ |
| There is a strong community of CHWs in Iowa | ☐ | ☐ | ☐ | ☐ | ☐ |
| **Supervision** |  |  |  |  |  |
| My supervisor values me as a member of the team | ☐ | ☐ | ☐ | ☐ | ☐ |
| My supervisor understands my job role. | ☐ | ☐ | ☐ | ☐ | ☐ |
| I am provided with adequate support to overcome the challenges I face in my work | ☐ | ☐ | ☐ | ☐ | ☐ |

| **Training** |  | | |  | | |  |
| --- | --- | --- | --- | --- | --- | --- | --- |
| The training I received when I started my job was adequate to help me succeed | ☐ | ☐ | ☐ | | ☐ | ☐ | |
| I have been provided with opportunities to enhance my skills through my work as a CHW | ☐ | ☐ | ☐ | | ☐ | ☐ | |
| I wish I had more training opportunities available to me | ☐ | ☐ | ☐ | | ☐ | ☐ | |

**The next set of questions refer to training you may have received at the beginning or throughout the course of your job as a CHW**

| When you started your current role as a CHW, were there any requirements for prior training, education, or other qualifications? | ☐ Yes | ☐ No | ☐ I don’t know |
| --- | --- | --- | --- |
| If yes, what were the requirements? |  |  |  |

|  |
| --- |
|  |

| When you started your current role as a CHW, were there any requirements for relevant experience? | ☐ Yes | ☐ No | ☐ I don’t know |
| --- | --- | --- | --- |
| If yes, approximately how much relevant experience was required? | ______________________ | |  |
| What was the relevant experience required? |  | |  |

|  |
| --- |
|  |

| Thinking about your current role as a CHW, have you received training in this role? | |  |
| --- | --- | --- |
|  | |  |
| ☐ | Yes – Internal (On the job training) | |
| ☐ | Yes - External (i.e. from a consulting company, conference, etc.) | |
| ☐ | Yes – Both internal and external | |
| ☐ | No, I received no training in this role | |
|  | If yes, approximately how many hours of training have you received? ____________________ | |

What skills did you receive training on when you started your current role? Please select all that apply.

| ☐ | N/A I did not receive training | ☐ | Interpersonal Communication |
| --- | --- | --- | --- |
| ☐ | Client Advocacy | ☐ | Interview strategies |
| ☐ | Counseling / Mentoring Techniques | ☐ | Leadership Training |
| ☐ | Cultural Competency | ☐ | Management / Organizational Skills |
| ☐ | First Aid / CPR | ☐ | Making Referrals |
| ☐ | Fundraising / Grant Writing | ☐ | Public Health Issues / Specific Disease Topics |
| ☐ | Health Education Methods | ☐ | Record Keeping / Data Management |
| ☐ | Health Coaching | ☐ | Safety |
| ☐ | Home visits | ☐ | Other (please specify): _____________ |

Is there any training that you have not received that you believe you would benefit from?

|  |
| --- |
|  |

| How is your work supervised? | |  | How is your work supervised? |  |
| --- | --- | --- | --- | --- |
|  | |  |  |  |
| ☐ | By Registered Nurses (RN’s) | | Yes – Internal (On the job training) | |
| ☐ | By another health professional (I.e. Physician, social worker, LPN, etc.) | | Yes - External (i.e. from a consulting company, conference, etc.) | |
| ☐ | By a member of administrative staff | | Yes – Both internal and external | |
| ☐ | By another Community Health Worker | | No, I received no training in this role | |
| ☐ | Other (please specify): _________________________ | | If yes, approximately how many hours of training have you received? ____________________ | |

| Does your supervisor have prior experience as a CHW? | ☐ Yes | ☐ No | ☐ Unsure |
| --- | --- | --- | --- |

| How is your performance monitored and evaluated? | |  | |
| --- | --- | --- | --- |
|  | |  | |
| ☐ | Monthly reviews | |  |
| ☐ | Annual reviews | |  |
| ☐ | Random skill evaluation | |  |
| ☐ | Continuing education sessions | |  |
| ☐ | No evaluation or monitoring | |  |
| ☐ | Other (please specify): ___________________________ | |  |

| Do you regularly document your encounters with clients? | ☐ Yes | ☐ No |
| --- | --- | --- |
| If yes, please provide a brief description of the documentation process: |  | |

|  |
| --- |

| Is there anything else you would like us to know about your experiences as a CHW that was not covered in this survey? |  |
| --- | --- |

|  |
| --- |

| Lastly, in an effort to aid us in reaching all individuals involved in CHW work, please list any organizations you think might benefit from engagement with this survey. |
| --- |

|  |
| --- |
|  |
|  |

**Supplementary Appendix D**

CHW and CHW employer interview

Codes and Sub-codes

In the following table, we have listed all the codes and sub-codes that came from the interviews with CHWs and CHW employers. For each code/subcode, we include a definition and provide one illustrative quote from a CHW interview, and another from an employer interview (unless the subcode is only specific to one or the other).

| **Code** | **Subcode** | **Definition** | **Illustrative Quotes (from CHW4 and E-CHW1)** |
| --- | --- | --- | --- |
| Motivation | CHW | Motivation of CHW to become a CHW | “I think naturally like my motivation was probably, when I graduated school, I wanted to go for social work. Didn't go for it. Went for marketing management and it just wasn't really what I was... I always wanted to know how can we be a part of the solution, so even when I had a young family, young mom, I always wanted to figure out... like, I can see things that I wanted to be better in. Instead of complaining about it, I always have the attitude of, well, how can I be a part of the solution. What can we do about it? It just fit really well with I already had worked with the schools, I worked with the churches, I got to know what was going on in the community before I had the job, so it was I kind of understood what those barriers were.” CHW1 |
|  | Employer – identified community need | Motivation of employer to hire CHWs based on needs in community | “So we saw them a lot of the Marshallese would just go to the emergency room because they didn't put their preventative care. It wasn't important. They didn't have the money for the visits. And so we really hired (CHW name) and the nurse to really do a lot of that education with the community.” E-CHW1 |
| Role/Responsibilities | Specific tasks | Primary role and/or responsibilities of the CHW focused on aspects other than SDoH | “we tend to do two units per year on specific healthcare topics where we're just working on spreading a very basic healthcare concept to as many people as possible. And then that goes in tandem with all of the navigation services. So we've done topics like when you call 911? What is the flu versus the common cold? What is mental health? Things like that..” E-CHW2 |
|  | SDoH | Primary role and/or responsibilities of the CHW focused on aspects related to any of the SDoH | “I will follow up like let's say somebody was struggling with food insecurity and I give them information on food pantries and applying for food assistance in WIC. And so I would follow up with them to see if they were able to connect with those services.” CHW4  “Yeah. We had a recent situation that a woman called us who was living in an apartment. She left a violent situation from (city outside Iowa), came to (city in Iowa), took the first place she found. The first week she was there realized that the apartment was infested with mice and bedbugs. And she called us wanting help. So the community worker went in, or worked with her and as he was trying to find housing, we became very much aware that subsidize housing in our local town.” E-CHW3 |
|  | Diverse Roles | Roles are broad, comprehensive | “the role of community health workers is as varied as the places that have them.” E-CHW3  “First of all, to know that there is a solution, there is a solution to the problem and also to offer some guidelines and some techniques which can help the person to, to, to to deal with the stress to deal with anxiety, to deal the depression. Also, it is not a medical, medical service but it offers a lot to a person because most of the people don't know that they are affected by it. I mean, by stress or whatever they see things not moving, but they don't know.” CHW5 |
|  | Advocate (for more complete understanding of clients lives) / Cultural broker | Role of CHW to advocate for clients | “CHW: Yeah. Usually like after I talk with a patient I let the provider know what's going on and you know that this is why they had missed maybe a child missed well-child exams. And so maybe transportation issues or they had to move. So in that sense, I advocate for the patient.  I: Yeah. So you kinda complete the picture, don't you?  ..  CHW: And you see no show, no show, no show as far as appointments and somebody can look at that and think, ‘Oh they think our healthcare isn't very important.’…..  But you can also look at it that what have they got going on in their lives that is causing them to not make appointments.” CHW4  “one of our health advocates was at a parent teacher conference and she was doing interpretation for a different program. And this mom had her little boy with her and that health advocate was like, "Something's wrong with your boy, I'm worried about him. "We need to take him to the hospital." And he actually had asthma. And by the time they got him to the hospital, he'd already passed out.” E-CHW2 |
|  | Building trust/connecting to community |  | “And another thing which is very important is to find a trusted the person of the community. For example, the immigrants, wanting to do a team plan and make it successful. You need somebody who can really reach out to many people who is accepted among the people. Not everyone can do (intervention program), because (intervention program) is like you are dealing with the life of somebody so someone should really trust you very much..” CHW5  “it's a little bit easier for us at (organization name) because our community healthcare workers are that community ally aspect of the process.” E-CHW2 |
|  | Pie in the sky (or some other term ☺) | Statements about the potential of CHW role to change lives | “In my role in my job I feel like my job is to give patients hope and to help them to be self-sufficient.” CHW4  “Yeah, yeah. And I think that makes you feel welcome too. There's not probably many places in (City) or Iowa in general that you can walk in and the room exit this way is in Marshallese, restroom is in Marshallese, COVID signs are in Marshallese.” E-CHW1 |
|  | Evolving | Mentions evolving role of the CHW | “My job two years ago was when it had changed from doing primarily developmental screening to helping patients with resources and social determinants of health and so that's when it started evolving and it's still evolving into that.”  “I think we're gonna be in the process because of what we've been seeing and the success and the buy-in with both populations. I believe we're going to be hiring a community health worker or our African-American population as well now.” E-CHW1  “From that, we grew over the last 12 years a lot. We were able to bring in... we moved two times to bigger spaces, we've added a food pantry, we've partnered with over probably 25 different agencies that come to this area that may not have storefront, like brick and mortar buildings.” CHW1 |
|  | Challenges to carrying out roles | Description of challenges to doing the CHW work | “When the community is hesitant. So COVID right now has been the main subject. So it was very hard to, how can I word this. For people to be okay with the vaccine and not just go with what they're hearing. So have them hear the facts, the truth and then have them make a decision on that. Not because they heard from who knows what. So that's hard because word of mouth in my community spreads fast. So by the time I get the information they already have like false information about that.” CHW2  “biggest barrier is really learning how to translate medical terms.” E-CHW2 |
| Method of communication |  | Ways that the CHW communicates with the client | “We have different means of communication. It just depends on what's easiest. We have email, phone call at our physical office, at our office, and then we also have a work cell that works really, really well for many of our clients in this area. They may only be able to text.” CHW1  “So we did go in the homes and administer some COVID vaccines to some of our Marshallese. We used to go to the homes a lot just to do reminders things. Just to drop off cards, saying a reminder of this or a reminder of that. Now we have almost all of our text messages, all in Marshallese as well.” E-CHW1 |
| Typical day tasks | Planned | Planned tasks on a typical day for the CHW | “So usually I get them, if there's a clinic, I'll try to spread the word help people set up appointments, or be there to help the Spanish speaking community translate items. I also work with the pick a better snack program that works with the schools. So I do translation for that and also facilitate the learning lessons. And we just started going out to the gardens. So we usually have garden dates every Tuesdays.” CHW2  “They've got a schedule that they know they're going to be there the third Wednesday from one to four, and they can schedule some time, but they can make a referral at any point and we'll follow up on it.” E-CHW3 |
|  | Spur of the moment | Spur of the moment tasks on a typical day | “Then also usually patients that need help with social determinants of health those are kind of spur the moment kind of things that patients are being seen for something else and a need is identified and so the doctor will come ask me if I can talk to them and help them with resources.” CHW4 |
|  | Not typical | Statements that the tasks often vary from day to day | “Well, each day is different for starters.” CHW4 |
| Location of the tasks |  | Place where the CHW carried out the tasks | “I usually meet them while they’re still here at the clinic and I’ll go into the exam room.” CHW4  “So we're meeting people where they're at. So potentially spending time at the VA office so that when people come in on certain days they can schedule appointments with them to get them connected to the community resources or food pantries.” E-CHW3 |
| Health care team | Coordination | Coordination or integrating of the CHW with other members of the healthcare team | “I: Do you go into the exam room then where the provider is and just talk with them right there or do they come to you or you set up a different time to talk or?  CHW: I usually meet them while they're still here at the clinic and I'll go into the exam room.  I: Nice so that warm handoff” CHW4  “Oh, I think they work closely obviously with the doctors and the nurses because they've got to do a lot of that explaining medication stuff to them lab results stuff with them. Obviously they cross over into all departments. They're not just in our medical department they're referring to our wellness center, they're referring to dental, they're referring to behavioral health.” E-CHW1 |
|  | Unique role | CHW play a unique role on the healthcare team | “I'm not clinical for thing. So that sets me apart and I am typically not... I deal more with the social stressors and social determinants of health.” CHW4  “That's not gonna be something that a provider or a mainstream medical organization can come into the community and say, "You need to get this booster shot." That's gonna be the community healthcare workers that are gonna be the ones convincing people to do that.” E-CHW2 |
|  | Valued role | CHW are valued/appreciated for the role they play on the healthcare team | “Well, what we heard from our, our coworkers, cause it's me and my coworker, (name), she helps the Congolese community. They've told us several times that they're so happy that we're here because they're able to communicate easier with those communities in this area.” CHW2  “But seeing huge value from a provider standpoint, providers love the program, the clinicians love the program. I think we all knew that clinicians wanted to address these things, but were worried to ask the questions about social needs in fear that they wouldn't have the time or resources or really the expertise to address them.” E-CHW4 |
|  | Clarity of role | Statement about whether the role of the CHW is clear. | “I understand what my job is and what my responsibilities are and I think for the most part everybody else here does also.” CHW4  “but we really have their roles defined pretty good as far as expectations like (CHW name) your dental. Period. The person with that, (CHW 2 name) is in charge of any kind of charity case things through the hospital. It's like, that's her gig. Everyone go through (CHW 2 name). So we really have it identified that you pretty much know who to go to or what.” E-CHW1 |
|  | Risk to clients of not having CHW | What would happen if CHW was not there | “From what I've experienced before helping people they don't get the services that they need they get left behind.” CHW2  “Right. And the sad part is a lot of times we're connecting them to services that are already there, they just aren't aware of them. So I think the whole community benefits when we're utilizing the services, as well as the individual for their own safety and health.” E-CHW3 |
| Communities served | General | General statement of communities served | “she helps the Congolese community. They've told us several times that they're so happy that we're here because they're able to communicate easier with those communities in this area.” CHW2  “(It) is a program which intends to help people who had, who are having psychosocial problems, resulting from their family life. Like immigrants, they have challenges related to adaptation in the area, in the new country.” CHW5  “work with multiple different problems, concerns, all sorts of different types of people, low-income, high-income, aged. We do actually 19 and up. We'll take referrals for anybody in 19 and up. So it's a diverse population.” E-CHW3 |
| Barriers | Client Barriers | Barriers to access to care for clients | “I: do you, thinking about that is there language barriers sometimes when you are working with families..  CHW: Definitely language barriers. We have a full time Spanish interpreter.” CHW4  “Have my community know who the health department is. They really don't know what the health department is and what they're here for. Since we're in the building with DHS you just think it's for Medicaid and food stamps only.” CHW2  “Yeah. I do have so many barriers. First of all, the online platform was a big challenge because people, this is something new and new to me I was using the word spout or some times calling and trying you also sometimes to go to visit them families. So what mediums are mainly because of the pandemic, that is one. And then of course, some do things like you want to look at me as if I'm having some mental issues or I am like mad. That's what comes to mind in the people in the minds of the people. So the more I talk the more you explain it to the client, what to them pass the more they feel comfortable and they see how needed it is. So the barrier is at the beginning and if someone builds trust, then it goes more helpful along the way.” CHW5 (mental health CHW that was doing psychosocial support online)  “a lot of the time families will sacrifice medical or sacrifice a prescription because they can't afford food that month.” E-CHW2 |
| Support needed by CHW | Connection to other CHWs | Statement that the CHW would value connections with other CHWs | “I would say connecting me with other CHWs would be important.” CHW4  “Also really interested and excited to just start engaging the community more and seeing how do we bring multiple health systems together to start engaging community resources and partnering better? I think we're already starting to do that a little bit more, becoming more connected with the other healthcare systems and finding out who my peers are. But how do we get all those community health workers talking and all of us organizations talking so we're not duplicating things, I think, is something that Iowa Department of Public Health has a huge role in, just as a conveyor of course of two. Those are the things, I guess, that are top of my mind today, but it'll probably change tomorrow.” E-CHW4 |
|  | Connection to others | Statement re other supportive connections needed (e.g. supervisor) | “Yes, my supervisor is, and it's happened several times where, oh this is happening in my community. What can I do? And my supervisor will be like, well what do you want to do? What do you think you should do? And we kind of talk about it. And then he's like, I think that's great. Let's do it.” CHW2  “Yeah. I mean, having supervisor is very important because sometimes you encounter a case like that one and you really needed a support because we are not, we are trained but we don't have too many skills in that you need someone who has more mental health school skills. So I was working hand in hand with my supervisor whenever I talk to my client first of all I heard it straight from my supervisor, what I have done or even before going into the client sometimes I was telling my supervisor that you know what more I have a meeting with my client. So tell me, okay, go.” CHW5 |
|  | Resources | Statement that resources are needed or Resources available to assist the CHW in their roles | “I think that we would have a lot of resources that are available that no one would know about or there would be no one accessing a lot of them, so the picture would look like... the snapshot of the needs would be very, very skewed because it's just... I always say like sometimes the government creates a resource and I call it a pretend resource because it's really hard to access. You know what I mean? Like, well, it's a resource, but I don't know anybody that's ever been able to access it.” CHW1  “we realized there's none that are for training refugee community healthcare workers. There were some programs and I know we spoke to one that was in (State) that was a community healthcare worker training designed to train refugee community workers, but they were for communities that had been in the United States for a really long time on higher education levels. So there was nothing for any of those communities who maybe hadn't gone through high school or just that lower literacy level.” E-CHW2  “We have a resource where you could... say you're getting rid of a couch; you would call me. I would match it up with a family that needed a couch; I would give you a tax receipt. It just works. We may have five beds that we need, and we would get five beds in that month. It would just, works out. I don't know how to explain it.” CHW1 |
| Perception of CHW role | Stressful | Statement that the work of CHWs can be stressful | “When I have a client that's calling me because they just had a seizure and they're at home and they have nobody for their kids, that makes me sad, or I have school call and they're like, "They put you down as their only contact for emergency for their kids." I'm like, that tells me we have to build this support system. That's doesn't feel good and that's going to be really hard to change their outcome if we don't start building relationships for them that are healthy.” CHW1  “I think she probably could have handled that without the added stress of the pandemic. I also think just, it's getting... And they can be very intensive at the beginning as you move through to try and get somebody to a point that they're safe or that they're willing to accept the help. And then there may be a lot to do all at once. And I think that's sometimes hard for some people to realize how much is going on and how intense it could be, and try to let that go at the end of the day, and then come back to it the next day. I think that was part of what was happening.” E-CHW3 |
|  | Sense of helplessness | Feelings that CHWs are not able to not able to respond to all needs | “families that are struggling and there's only so much that I can do.” CHW4 |
|  | Satisfying | Feeling of satisfaction that comes from being a CHW | “When I help someone and they say, oh, thank you. I didn't know I could get that help. And I'm so glad you're there, having someone that speaks their language.” CHW2  “Yeah, well that is the practical or the practical part of (intervention program) when we started to practice a lot of fun. But when you are still doing the theories you're learning this, is like, Hmm, am I going to do something? But if applied it to real life experience to you. You'll see what you are, you enjoy what you are doing because you changed the life of a person. And when you are committed to it, for sure. Yeah. It makes a big difference.” CHW5 |
| COVID |  | Any mention of COVID in relation to the work or impact of the work of the CHW | “it's very important and the thing is that it's always changing, resources are always changing. keep something that's current is challenging to do. And especially with COVID--Our programs change how they did things.”  “So they do a lot of translating those forms for us as well. And various things come up. Cause you're always coming up with new forms, especially during COVID we had new signs going up all over the place for everything.” (E-CHW1)  “Right now with COVID not being able to go out to the community and when there's nothing else I can do, I feel like sometimes I have to solve the whole everybody's problem even though I know I can't, that's my least favorite part and not be able to help more.” CHW2 |
| Training | General | Acknowledgement of the importance of training for CHW | “I have to do culture diversity, I have to do... I just look at my emergency management, they do different trainings that we have to do. Between the hospital, what they expect of me, and then the agencies that I work with, I'm always looking for those types of things. Crisis intervention, and I kind of have a list and then, what ones are the most important, which ones I feel were really beneficial, and then, as the different needs come and go, I look for where my weaknesses are. Where... boy, this is a new need.” CHW1 |
|  | Competencies | List of specific training competencies for CHWs | “It taught me about cultural humility, motivational interviewing, public health in general, public health issues.” CHW4  “As you go they have to add the trainings like anybody else, like if they're a new hire here, any new hire paperwork, most of them have like the mandatory child abuse reporter. It's really teaching and they have to be in there electronic medical record.” E-CHW1 |
|  | Gaps | Statement of training needs/gaps for CHWs | “We feel like we're really good at understanding poverty, but we really need a refresher probably because especially when that [inaudible 00:41:29] a lot of times serving.” CHW1  “the biggest barrier is really learning how to translate medical terms. Cause it's one thing to translate. I mean you go to the doctor sometimes and they're speaking English and you don't know what they're saying. So really not being afraid to look at the provider and say we have no idea what you're talking about. Like, I don't even understand that in English, let alone trying to translate it in Marshallese, especially with the Marshallese language, there's just certain words they just don't think exists. I mean, they don't exist. So those struggles with just medical language in general can be hard.” E-CHW1 |
|  | For Providers other than CHW | Training needed for providers | “And we do a lot with our providers how to use translators, when to pause, do more descriptives, don't say stuff like psychosocial development, third grade language here folks because we are trying to interpret it to a different language that won't always translate smoothly.” E-CHW1  “when we train our team, we always talk about, you're a cultural bridge between your client and a provider but you're also a cultural bridge for that provider to the client, so it goes both ways.” E-CHW2 |
| Recruitment of more CHWs | Barriers | Barriers that impeded growth of jobs as CHWs | “I: If somebody in the community wanted to become a CHW what would be the barriers? .. CHW4  CHW: I think it's probably a lack of awareness of CHW jobs and what that entails 'cause that still fairly new to Iowa.” CHW4  “There's a whole plethora of things that make it really challenging. And as community healthcare workers are more discussed, it also becomes more streamlined. And a lot of those programs are becoming credentialed which then prevents our community healthcare workers from being formally recognized. And that's a huge dilemma and barrier for our team, because if they're not formally recognized as doing that work then they don't get that opportunity to work with the medical professionals, like I mentioned earlier and really eliminate some of those barriers.” E-CHW2 |
|  | Approaches to recruiting | Description of approaches to recruiting (e.g. networking, fit the need of the community) | “when we go to recruit a lot of the time it's from our own team. So when we have, for example, if we're recruiting navigators, a lot of the time we're talking to our existing staff and saying, "Who in your community is passionate about this "and would be interested in learning more about this." And then they come on and their first phase is navigator. Now, if they really love being a navigator and it's something they're passionate about then we'll talk about training them from our own staff and having them become an advocate. So it's kind of, it's more like a ladder stuff. We actually have really excitingly, had a lot of our team members go on from being advocates, working at other medical facilities.” E-CHW2 |
| Documentation of work with clients |  | Methods of documenting the work that is being done by CHWs | “Yeah, that's exactly what I do. It's usually connected with the... Like if the doctor saw the patient and then asked me to go in and see the patient with resources, my note would be connected to the doctor's note and if I follow up or when I follow up then I would do it like a telephone note…… I don’t use anything really outside of the health record.” CHW4 |
| Funding | General | Funding for CHW program | “And as we looked at funding to continue that we reached out with one of our partners on the care transitions who connected us closer to public health. And so public health has funded half of our program for the first year. And with that we kind of had to change our role that we were looking at because their goal was that within the county we would look at people that the fire department, the police, the EMS went out to that were frequent flyers, or they were out there and realized there were more problems, and how could they make a one call and be done to follow up on things afterwards? So, that's more the route that we've ended up taking with our community worker. And then we're looking at different sites. So they were stalled with COVID, things kind of put a damper on it.” E-CHW3  “We create groups and support groups, and we'd get tons of funding from the community. They would endowments and then donations because we would help. I just always had... I'd always tell the people that we worked with that are even our volunteers, if we do things for the right reason, it will just work out, and it just does.” CHW1 |
| Success | Story | Sharing a success story of the impact of the CHW or a CHW program | “When I first started, because it was around COVID time. In October of last year we also got trained to do contact tracing. So I I called a Spanish speaking family for contact tracing. And I can't remember exactly but it went from contact tracing to me, helping her. She had just had a baby. Her baby was in Iowa city. Her husband lost her job, his job. She lost her job. Her baby was premature. And they didn't know if the baby was going to make it. She had a stack of bills that she didn't know what to do with the hospital bills. She didn't know if her baby had Medicaid. She didn't know she could get with food for the moment. And then she had just gotten her before that, her permit. So she, she didn't know how to get her license from the permit to a license. And then she had to get an appointment. So I helped her figure out the bills, Medicaid, her license. And then I was able to help her get connected to WIC to get WIC. And from there, she just calls me now.” CHW2 |
|  | Indicator of success | Indicators of success (referrals, reputation, partnerships, community awareness and buy-in, etc) | "You know what? "It's been so nice to have one of your team "in our office with this client "because there's things for example "I wouldn't know when they're having Ramadan, for example." E-CHW2  “we were getting called from the homeless shelters from the schools, from the court systems for everybody else that needed help with everything and housing and all of that stuff.” E-CHW1 |
|  | Pride in program | Feelings of pride in the CHW program | “Yeah. We're doing a pretty good job with primary care. I would say in the North Iowa .. region, we're universally screening for social needs at all of primary care, and have community health workers attached to those clinics. Community health workers in those regions, they're supporting multiple clinics though. Then in the (city in Iowa) we probably have about half of primary care covered. We've added some really nice efficiencies in the past year with the screening process and some data integration and things like that. Less manual entry and things like that, so we've been able to expand there. We have one community health worker serving pediatric population. We were able to start that with a grant through (Foundation), so that was great.” E-CHW4 |
| Qualities of successful CHWs |  | Qualities that make a successful CHW (leader, connected to/from community, community trust, language/interpreting, etc) | “..outside of her role here at (Organization name), she really is a female strong leader in that whole community. ... And she just knows everybody, people trust her people believe in her.” E-CHW1  “Well, you needed to be resilient… When you're dealing with a person who sometimes doesn't know that he has a problem or someone takes for granted that this is a problem, but it is not a serious problem or whatever, but I mean, you have to be resilient. You don't need to get discouraged.” CHW5 |
| Qualities of unsuccessful CHWs |  | Qualities that hinder success of a CHW | “at the end of the day somebody who doesn't have any of skills and tends to be very, like linear and non-explanatory with things, that person's not quite as good as a community healthcare worker,” E-CHW2 |
| Cultural referencing |  | Referring to aspects of culture that affect CHW role or approach | “Like with (name of CHW), we really knew that from what(name of CHW2) was telling us, not all the men in the Marshallese community were real comfortable with (name of CHW2) providing the interpretation when it might've been some sensitive male issues. So it was very deliberate when we hired (name of CHW) that we wanted a male for that role.” E-CHW1  “And then her, she is from Guatemala. So her first language is not Spanish. It's her own native language from her tribe. So that would, that even makes it harder for her.” CHW2 |
| CHW **programmatic** | Evolution | How the CHW program evolved | “It was just a very small space that was, like I said, one employee, very minimal hours, but then because we're in a very rural area, it didn't take long for that person to identify that domestic violence advocate and the fuel assistance with community action, were also looking for a place to land because our county is so large when they were in this area. With this space that was a very small office, with a couple little... actually, it was a couple of small offices together, we created this partnership, and we could utilize space and create this collaboration.” CHW1  “We've had CHWs, we started them probably 18 months ago. And it might've been a little longer than that. We've been working on different pieces of it for about two years and we've had our care transitions program, they started in 2012. So we've gone from working with a hospital to we now do a return to community through the Iowa (gov, agency) with some funding and a trial that they're doing in three of our counties. So, and that just evolved over time the last 10 years, and the community health worker was just a good fit to bring in.” E-CHW3 |
|  | Lessons learned | Lessons learned programmatically | “I learned a long time ago I have to do a lot for places, for those programs, to get... and that's how we built the relationship, so maybe I have to be on your board, or I have to come to your... and that's fine. We'll do that because it makes me learn, it gives me the opportunity to know more and learn more about how that actually works. Then, I can give a more clear perspective on what exactly from the client, as well as from you, where the breakdown is.” CHW1  “I think we have changed. I think we've done stuff that wasn't successful and we've learned and so we've tried different things. So obviously when we first started this we've made changes to it that and it's been an ever growing changes. What makes sense and with the addition I'm going why not have you guys just fill out the Medicaid applications, or why can't we just send out the passports It doesn't seem so difficult teaching ourselves but that would've been something that (CHW name) just wouldn't have been able to do that (another CHW name) can do. And in that roles and early on we were learning that, people weren't getting the diabetic education they weren't learning, they we weren't seeing changes that someone does. They're very group oriented activity let's run a group session on diabetic education ended up being hugely successful.” E-CHW1 |
|  | Programmatic strategy | What are the strategies that the CHW program implements? (e.g. work through community leaders / adapt to community, etc) | “I think just like being able to cut through all that red tape and just like, let's try it, let's monitor it, let's pilot... I always call it pilot, a pilot program. We've done so many pilot programs like that. We're just finishing up an eight week... it's called (intervention program). It's a program that we did with the therapists and with... well, and a yoga instructor, a massage therapist. It's kind of both because we needed both of those naturalistic kind of things and the professional side. They facilitated an eight week course. I had instantly 15 people sign up and a wait list, and they love it. They are so upset that it's ending, and I just think like... but you know what it did? It created those friendships. It created that level of how do... The reason why we did it is we needed to get them to make connections. It's that connections that we talked about.” CHW1  “a lot of our programs support families as a whole, or our work with youth. We have some youth programs, we know when there's younger individuals coming up through the generation, maybe they're in school for nursing. Maybe they have their family has had a service from (CBO) where we've helped them with some sort of navigation.” E-CHW2 |

**Appendix E:**

|  | **Standards for Reporting Qualitative Research (SRQR)*** |  |
| --- | --- | --- |
|  |  | **Page/line no(s).** |
| **Title and abstract** | |  |
|  | **Title** - Concise description of the nature and topic of the study Identifying the study as qualitative or indicating the approach (e.g., ethnography, grounded theory) or data collection methods (e.g., interview, focus group) is recommended | Title |
|  | **Abstract** - Summary of key elements of the study using the abstract format of the intended publication; typically includes background, purpose, methods, results, and conclusions | Page 2 |
|  |  |  |
| **Introduction** | |  |
|  | **Problem formulation** - Description and significance of the problem/phenomenon studied; review of relevant theory and empirical work; problem statement | Page 3-4 |
|  | **Purpose or research questio**n - Purpose of the study and specific objectives or questions | Page 4, lines 105-109 |
|  |  |  |
| **Methods** | |  |
|  | **Qualitative approach and research paradigm** - Qualitative approach (e.g., ethnography, grounded theory, case study, phenomenology, narrative research) and guiding theory if appropriate; identifying the research paradigm (e.g., postpositivist, constructivist/ interpretivist) is also recommended; rationale** | Page 5, lines 148-154 |
|  | **Researcher characteristics and reflexivity** - Researchers’ characteristics that may influence the research, including personal attributes, qualifications/experience, relationship with participants, assumptions, and/or presuppositions; potential or actual interaction between researchers’ characteristics and the research questions, approach, methods, results, and/or transferability | Page 5, 139-141 |
|  | **Context** - Setting/site and salient contextual factors; rationale** |  |
|  | **Sampling strategy** - How and why research participants, documents, or events were selected; criteria for deciding when no further sampling was necessary (e.g., sampling saturation); rationale** | Page 5, 133-139 |
|  | **Ethical issues pertaining to human subjects** - Documentation of approval by an appropriate ethics review board and participant consent, or explanation for lack thereof; other confidentiality and data security issues | Page 4-5, lines 107-108, 143-144 |
|  | **Data collection methods** - Types of data collected; details of data collection procedures including (as appropriate) start and stop dates of data collection and analysis, iterative process, triangulation of sources/methods, and modification of procedures in response to evolving study findings; rationale** | Page 4-5, lines 126-158 |
|  | **Data collection instruments and technologies** - Description of instruments (e.g., interview guides, questionnaires) and devices (e.g., audio recorders) used for data collection; if/how the instrument(s) changed over the course of the study | Page 4, lines 126-132, 144-145 |
|  | **Units of study** - Number and relevant characteristics of participants, documents, or events included in the study; level of participation (could be reported in results) | Page 5, lines 168-170, Table 1 |
|  | **Data processing** - Methods for processing data prior to and during analysis, including transcription, data entry, data management and security, verification of data integrity, data coding, and anonymization/de-identification of excerpts | Page 5, lines 143-147 |
|  | **Data analysis** - Process by which inferences, themes, etc., were identified and developed, including the researchers involved in data analysis; usually references a specific paradigm or approach; rationale** | Page 5, lines 148-158 |
|  | **Techniques to enhance trustworthiness** - Techniques to enhance trustworthiness and credibility of data analysis (e.g., member checking, audit trail, triangulation); rationale** | Page 5, lines 148-155 |
|  |  |  |
| **Results/findings** | |  |
|  | **Synthesis and interpretation** - Main findings (e.g., interpretations, inferences, and themes); might include development of a theory or model, or integration with prior research or theory | Pages 6-8, Appendix D |
|  | **Links to empirical data** - Evidence (e.g., quotes, field notes, text excerpts, photographs) to substantiate analytic findings | Pages 6-8, Table 5, Appendix D |
|  |  |  |
| **Discussion** | |  |
|  | **Integration with prior work, implications, transferability, and contribution(s) to the field -** Short summary of main findings; explanation of how findings and conclusions connect to, support, elaborate on, or challenge conclusions of earlier scholarship; discussion of scope of application/generalizability; identification of unique contribution(s) to scholarship in a discipline or field | Page 11-12, lines 414-445,462-478 |
|  | **Limitations** - Trustworthiness and limitations of findings | Page 12, 448-460 |
|  |  |  |
| **Other** | |  |
|  | **Conflicts of interest** - Potential sources of influence or perceived influence on study conduct and conclusions; how these were managed | Page 13 |
|  | **Funding** - Sources of funding and other support; role of funders in data collection, interpretation, and reporting | Page 13 |
|  |  |  |
|  | *The authors created the SRQR by searching the literature to identify guidelines, reporting standards, and critical appraisal criteria for qualitative research; reviewing the reference lists of retrieved sources; and contacting experts to gain feedback. The SRQR aims to improve the transparency of all aspects of qualitative research by providing clear standards for reporting qualitative research. |  |
|  |  |  |
|  | **The rationale should briefly discuss the justification for choosing that theory, approach, method, or technique rather than other options available, the assumptions and limitations implicit in those choices, and how those choices influence study conclusions and transferability. As appropriate, the rationale for several items might be discussed together. |  |
|  |  |  |
|  | **Reference:** |  |
|  | O'Brien BC, Harris IB, Beckman TJ, Reed DA, Cook DA. **Standards for reporting qualitative research: a synthesis of recommendations.** *Academic Medicine*, Vol. 89, No. 9 / Sept 2014  DOI: 10.1097/ACM.0000000000000388 |  |
|  |  |  |
|  |  |  |

**Checklist for Reporting Of Survey Studies (CROSS)**

| **Section/topic** | **Item** | **Item description** | **Reported on page #** |
| --- | --- | --- | --- |
| **Title and abstract** | | |  |
| Title and abstract | 1a | State the word “survey” along with a commonly used term in title or abstract to introduce the study’s design. | Page 2, line 27 |
|  | 1b | Provide an informative summary in the abstract, covering background, objectives, methods, findings/results, interpretation/discussion, and conclusions. | Page 2 |
| **Introduction** | | |  |
| Background | 2 | Provide a background about the rationale of study, what has been previously done, and why this survey is needed. | Page 3-4 |
| Purpose/aim | 3 | Identify specific purposes, aims, goals, or objectives of the study. | Page 4 |
| **Methods** | | |  |
| Study design | 4 | Specify the study design in the methods section with a commonly used term (e.g., cross-sectional or longitudinal). | Page 4-5, lines 113,119,161 |
|  | 5a | Describe the questionnaire (e.g., number of sections, number of questions, number and names of instruments used). | Page 5, lines 163-167 |
| Data collection methods | 5b | Describe all questionnaire instruments that were used in the survey to measure particular concepts. Report target population, reported validity and reliability information, scoring/classification procedure, and reference links (if any). | N/A |
|  | 5c | Provide information on pretesting of the questionnaire, if performed (in the article or in an online supplement). Report the method of pretesting, number of times questionnaire was pre-tested, number and demographics of participants used for pretesting, and the level of similarity of demographics between pre-testing participants and sample population. | N/A |
|  | 5d | Questionnaire if possible, should be fully provided (in the article, or as appendices or as an online supplement). | Supplementary Appendix C |
| Sample characteristics | 6a | Describe the study population (i.e., background, locations, eligibility criteria for participant inclusion in survey, exclusion criteria). | Page 5, lines 167-171 |
|  | 6b | Describe the sampling techniques used (e.g., single stage or multistage sampling, simple random sampling, stratified sampling, cluster sampling, convenience sampling). Specify the locations of sample participants whenever clustered sampling was applied. | Page 5, lines 172-175, 180-181 |
|  | 6c | Provide information on sample size, along with details of sample size calculation. | Page 6, 183-186 |
|  | 6d | Describe how representative the sample is of the study population (or target population if possible), particularly for population-based surveys. | Page 6, 172-175 |
| Survey  administration | 7a | Provide information on modes of questionnaire administration, including the type and number of contacts, the location where the survey was conducted (e.g., outpatient room or by use of online tools, such as SurveyMonkey). | Page 5, lines 172-181 |
|  | 7b | Provide information of survey’s time frame, such as periods of recruitment, exposure, and follow-up days. | Page 5, line 179-180 |
|  | 7c | Provide information on the entry process:  –>For non-web-based surveys, provide approaches to minimize human error in data entry.  –>For web-based surveys, provide approaches to prevent “multiple participation” of participants. | Page 6, line 190-193 |
| Study preparation | 8 | Describe any preparation process before conducting the survey (e.g., interviewers’ training process, advertising the survey). | Page 4, lines 113-119 |
| Ethical considerations | 9a | Provide information on ethical approval for the survey if obtained, including informed consent, institutional review board [IRB] approval, Helsinki declaration, and good clinical practice [GCP] declaration (as appropriate). | Page 4, line 122-124 |
|  | 9b | Provide information about survey anonymity and confidentiality and describe what mechanisms were used to protect unauthorized access. | Page 5-6, lines 181-183 |
| Statistical  analysis | 10a | Describe statistical methods and analytical approach. Report the statistical software that was used for data analysis. | Page 6 lines 187-193 |
|  | 10b | Report any modification of variables used in the analysis, along with reference (if available). | N/A |
|  | 10c | Report details about how missing data was handled. Include rate of missing items, missing data mechanism (i.e., missing completely at random [MCAR], missing at random [MAR] or missing not at random [MNAR]) and methods used to deal with missing data (e.g., multiple imputation). | Page 6, line 191-193 |
|  | 10d | State how non-response error was addressed. | Page 6, line 187-188 |
|  | 10e | For longitudinal surveys, state how loss to follow-up was addressed. | N/A |
|  | 10f | Indicate whether any methods such as weighting of items or propensity scores have been used to adjust for non-representativeness of the sample. | N/A |
|  | 10g | Describe any sensitivity analysis conducted. | N/A |
| **Results** | | |  |
| Respondent characteristics | 11a | Report numbers of individuals at each stage of the study. Consider using a flow diagram, if possible. | Page 8, line 280-282; Page 9, line 338-340 |
|  | 11b | Provide reasons for non-participation at each stage, if possible. | N/A |
|  | 11c | Report response rate, present the definition of response rate or the formula used to calculate response rate. | Page 6, line 187-188 |
|  | 11d | Provide information to define how unique visitors are determined. Report number of unique visitors along with relevant proportions (e.g., view proportion, participation proportion, completion proportion). | Page 8, line 280-282; Page 9, line 338-340 |
| Descriptive  results | 12 | Provide characteristics of study participants, as well as information on potential confounders and assessed outcomes. | Table 2 |
| Main findings | 13a | Give unadjusted estimates and, if applicable, confounder-adjusted estimates along with 95% confidence intervals and p-values. | N/A |
|  | 13b | For multivariable analysis, provide information on the model building process, model fit statistics, and model assumptions (as appropriate). | N/A |
|  | 13c | Provide details about any sensitivity analysis performed. If there are considerable amount of missing data, report sensitivity analyses comparing the results of complete cases with that of the imputed dataset (if possible). | N/A |
| **Discussion** | | |  |
| Limitations | 14 | Discuss the limitations of the study, considering sources of potential biases and imprecisions, such as non-representativeness of sample, study design, important uncontrolled confounders. | Page 12, lines 448-460 |
| Interpretations | 15 | Give a cautious overall interpretation of results, based on potential biases and imprecisions and suggest areas for future research. | Pages 11-12 |
| Generalizability | 16 | Discuss the external validity of the results. | Pages 11-12 |
| **Other sections** | | |  |
| Role of funding source | 17 | State whether any funding organization has had any roles in the survey’s design, implementation, and analysis. | Page 13 |
| Conflict of interest | 18 | Declare any potential conflict of interest. | Page 13 |
| Acknowledgements | 19 | Provide names of organizations/persons that are acknowledged along with their contribution to the research. | Page 13 |
